# Antifungal Drug Concentration Impacts the Spectrum of Adaptive Mutations in *Candida albicans*

**DOI:** 10.1093/molbev/msad009

**Published:** 2023-01-17

**Authors:** Robert T Todd, Natthapon Soisangwan, Sam Peters, Bailey Kemp, Taylor Crooks, Aleeza Gerstein, Anna Selmecki

**Affiliations:** Department of Microbiology and Immunology, University of Minnesota Medical School, Minneapolis, Minnesota; Department of Microbiology and Immunology, University of Minnesota Medical School, Minneapolis, Minnesota; Bioinformatics and Computational Biology Graduate Program, University of Minnesota, Minneapolis, Minnesota; Department of Microbiology and Immunology, University of Minnesota Medical School, Minneapolis, Minnesota; Department of Microbiology and Immunology, University of Minnesota Medical School, Minneapolis, Minnesota; Department of Microbiology and Immunology, University of Minnesota Medical School, Minneapolis, Minnesota; Department of Microbiology, The University of Manitoba, Winnipeg, Manitoba, Canada; Department of Statistics, The University of Manitoba, Winnipeg, Manitoba, Canada; Department of Microbiology and Immunology, University of Minnesota Medical School, Minneapolis, Minnesota; Bioinformatics and Computational Biology Graduate Program, University of Minnesota, Minneapolis, Minnesota

**Keywords:** copy number variation, aneuploidy, polyploidy, antifungal drug resistance, antifungal drug tolerance, *Candida albicans*

## Abstract

Invasive fungal infections are a leading global cause of human mortality. Only three major classes of antifungal drugs are widely used, and resistance to all three classes can arise rapidly. The most widely prescribed antifungal drug, fluconazole, disseminates rapidly and reaches a wide range of concentrations throughout the body. The impact of drug concentration on the spectrum and effect of mutations acquired during adaptation is not known for any fungal pathogen, and how the specific level of a given stress influences the distribution of beneficial mutations has been poorly explored in general. We evolved 144 lineages from three genetically distinct clinical isolates of *Candida albicans* to four concentrations of fluconazole (0, 1, 8, and 64 μg/ml) and performed comprehensive phenotypic and genomic comparisons of ancestral and evolved populations. Adaptation to different fluconazole concentrations resulted in distinct adaptive trajectories. In general, lineages evolved to drug concentrations close to their MIC_50_ (the level of drug that reduces growth by 50% in the ancestor) tended to rapidly evolve an increased MIC_50_ and acquired distinct segmental aneuploidies and copy number variations. By contrast, lineages evolved to drug concentrations above their ancestral MIC_50_ tended to acquire a different suite of mutational changes and increased in drug tolerance (the ability of a subpopulation of cells to grow slowly above their MIC_50_). This is the first evidence that different concentrations of drug can select for different genotypic and phenotypic outcomes in vitro and may explain observed in vivo drug response variation.

## Introduction

The evolution of antifungal drug resistance is a growing global health concern. Invasive fungal infections caused by opportunistic and recently emerged fungal pathogens are associated with high mortality rates and increased healthcare-associated costs, particularly in immunocompromised patients (Patterson 2002; Pfaller 2012; Pfaller et al. 2019). Over 72 million invasive fungal infections are identified globally per year (Pfaller et al. 2019). *Candida albicans* is the most prevalent causative agent of invasive fungal infections globally and the mortality rate of these infections is incredibly high (20–60%) despite modern antifungal drug treatment regimens (Pfaller et al. 2010; Andes et al. 2016; Pfaller et al. 2019). This antifungal treatment failure is attributed to the remarkable ability of *C. albicans* to colonize and adapt to diverse niches and antifungal drug concentrations within the host.

*Candida albicans* is a heterozygous diploid yeast that has a labile genome and exhibits extensive intra-species genetic diversity with an average nucleotide diversity between any two isolates of ∼0.37% (Selmecki et al. 2006; Ford et al. 2015; Hirakawa et al. 2015; Ropars et al. 2018). Although there is some evidence of limited meiotic recombination (Ropars et al. 2018), the vast majority of genetic diversity is generated via asexual mitotic recombination (Bennett and Johnson 2003; Lephart and Magee 2006; Forche et al. 2011, 2018; Wang et al. 2018). In addition to single-nucleotide variants (SNVs), frequent karyotypic differences including aneuploidy, polyploidy, inversions, and loss of heterozygosity (LOH) exist between clinical isolates. These large-scale genome changes also arise during in vitro and in vivo evolution experiments and have major implications on how *Candida* adapts to new or stressful environments (Suzuki et al. 1982; Rustchenko-Bulgac 1991; Chibana et al. 2000; Magee and Magee 2000; Selmecki et al. 2006; Forche et al. 2011, 2018; Hickman et al. 2013; Ford et al. 2015; Hirakawa et al. 2015; Todd et al. 2019; Todd and Selmecki 2020; Wang et al. 2021; Kukurudz et al. 2022). Up to ∼20% of cells in a population can become polyploid in as little as 8 h when cultured in 10 μg/ml FLC due to multipolar spindle formation and/or cytokinesis failure (Harrison et al. 2014). These polyploidization events can occur in different fungal species and diverse *C. albicans* genetic backgrounds (Harrison et al. 2014; Gerstein and Berman 2020). However, the impact of drug concentration on frequency of polyploidization events and the competitive fitness of polyploid cells over short and long timescales in FLC, remain poorly understood (Gerstein and Sharp 2021).

Only three major classes of antifungal drugs have been approved for use in humans: echinocandins, polyenes, and azoles. Globally, the azole drug fluconazole (FLC) is the most frequently prescribed antifungal drug due to its high bioavailability, low cost, and ease of administration. Azole drugs are fungistatic and inhibit the biosynthesis of ergosterol and cause severe membrane stress to the fungal cell. FLC exhibits linear pharmacokinetics and excellent distribution into various tissues and body fluids, reaching a broad range of physiological concentrations. Serum concentrations are dose-dependent, with typical doses of 200–800 mg/day FLC corresponding to serum concentrations of ∼7.5–60.5 μg/ml FLC (Schiave et al. 2018). FLC levels reach peak serum concentration 1–6 h after administration and remain in the system for days (elimination half-life is ∼30 h in individuals with functioning kidneys [Cousin 2003]). Additionally, FLC concentrations vary between different tissues in the body (1 μg/ml to over 22 μg/ml), with the highest concentration of FLC being detected in the spleen (Fischman et al. 1993). Lower doses of FLC are used as prophylaxis for patients undergoing solid tissue transplantation (Felton et al. 2014; Pappas et al. 2016; Schiave et al. 2018) and recurrent vulvovaginal candidiasis (Denning et al. 2018), however higher daily doses of FLC may result in longer survival of patients with candidiasis (Schiave et al. 2018). Why different drug concentration influence treatment success remains largely unknown.

Azole drug resistance arises in clinical settings during antifungal therapy and severely limits subsequent treatment options. Drug susceptibility is determined in the laboratory at 24 h and defined as the minimum inhibitory concentration (MIC) that reduces 50% of growth (MIC_50_), and drug resistance is the ability to grow in drug concentrations that inhibit susceptible isolates, typically defined by epidemiological cut-off values (Pfaller et al. 2010, 2011). Known mechanisms of azole resistance include the upregulation of drug efflux pumps (ABC family and MFS multidrug efflux pumps), mutations in the gene encoding the azole drug target, *Erg11,* and mutations that bypass the membrane stress response (Marr et al. 1998; Cowen et al. 2000; Anderson et al. 2003, 2004; Selmecki et al. 2006; Bouchonville et al. 2009; Fothergill et al. 2014; Harrison et al. 2014; Ford et al. 2015; Todd et al. 2019; Todd and Selmecki 2020). Additionally, ∼50% of all FLC-resistant *C. albicans* isolates contain at least one aneuploid chromosome (Selmecki et al. 2006) and chromosomal instability, in general, can increase the frequency of FLC-resistant cells (Brimacombe et al. 2019; Yang, Todd, et al. 2021). One specific segmental aneuploidy, isochromosome 5L (i(5L)), causes drug resistance via an increase in the copy number of *ERG11* and *TAC1* (Selmecki et al. 2006, 2008). i(5L) can arise rapidly under FLC selection, occurs in diverse genetic backgrounds of *C. albicans*, and frequently results in multi-azole resistance (Selmecki et al. 2008, 2009).

In addition to azole resistance, azole tolerance (defined as the ability of a drug-susceptible isolate to grow slowly in drug concentrations above the MIC_50_ beyond 24 h) also has major implications in clinical settings for fungistatic drugs that metabolically inhibit, rather than kill, susceptible cells (Berman and Krysan 2020). Fungal drug tolerance is linked to azole treatment failure and the inability to clear an infection, despite these isolates being considered drug-sensitive by the typical MIC_50_ assays (Sanglard et al. 2003; Delarze and Sanglard 2015; Rosenberg et al. 2018; Berman and Krysan 2020). Currently, it is thought that antifungal drug tolerance occurs via enhanced signaling of cellular stress response pathways including modulation of the calcium signaling pathway, nutrient detection, and *HSP90* activation (Cowen and Steinbach 2008; Rosenberg et al. 2018; Murphy and Bicanic 2021). Drug-tolerant fungal cells are often present as a distinct subpopulation and can evolve independently of drug resistance, existing alongside drug-resistant or susceptible cells (Gerstein and Sethi 2022). Tolerance in fungi seems to be a stable characteristic and isolates that evolve increased tolerance do not change in the prolonged absence of FLC (Rosenberg et al. 2018). Notably, tolerance has distinct definitions in the bacterial and fungal communities (Westblade et al. 2020; Michaux et al. 2022). As an example, FLC-tolerant *Candida* do not exhibit the slowed growth phenotype that tolerant bacterial cells exhibit in the absence of drug (Rosenberg et al. 2018; Todd and Selmecki 2020; Michaux et al. 2022). Intriguingly, our recent experiments demonstrate that azole tolerance and cross-tolerance to different azole drugs can evolve rapidly in vitro (Todd and Selmecki 2020; Kukurudz et al. 2022), yet the mechanisms that distinguish drug-resistant and drug-tolerant phenotypes in fungi are not fully characterized.

Experimental evolution provides an opportunity to pinpoint the order of events leading to antifungal drug resistance and drug tolerance, the degree of parallelism among adaptive mutations between different genetic backgrounds and different environments, and the phenotypic effect of mutations acquired during adaptation. Prior studies into how antifungal drugs impact the spectrum of mutations observed during adaption primarily focused on increasing FLC concentrations or on short-term evolution experiments in a single concentration of FLC (Cowen et al. 2000; Anderson et al. 2003; Mount et al. 2018; Rybak et al. 2020; Burrack et al. 2022). In one example, six lineages from one *C. albicans* strain were maintained at increasing concentrations of FLC for 300–400 generations, where the MIC_50_ was quantified every 10–20 generations and the drug concentration was increased to twice the MIC_50_, to a maximum concentration of 128 μg/ml FLC (Cowen et al. 2000). In a similar example, three lineages from one genetic background of haploid *S. cerevisiae* were exposed to increasing concentrations of FLC over the span of 400 generations, starting at 16 μg/ml FLC and increasing every 100 generations to 32 μg/ml, 64 μg/ml, and 128 μg/ml FLC (Anderson et al. 2003). Although these and other evolutionary studies provided valuable insight into mechanisms that drive azole resistance, little is known about how prolonged exposure to a constant drug concentration impacts evolutionary trajectories and what distinguishes selection for azole resistance from azole tolerance.

Overall, the impact of genetic and environmental factors, including FLC concentration, on the acquisition of drug resistance and tolerance phenotypes is not known. Here, we provide the first comprehensive analysis of how three different clinical isolates of *C. albicans* adapt to different physiological concentrations of FLC using a single standardized methodology. We characterized the phenotypic responses and mutational spectrum (single-nucleotide polymorphisms, aneuploidy, segmental aneuploidy, and whole-genome ploidy changes) at four different drug concentrations during adaptation of *C. albicans* in vitro. We first identified the spectrum and effect of mutations acquired in two different genetic backgrounds with the same starting MIC (0.5 μg/ml) and found that drug concentrations near the starting MIC selected for increases in the frequency of segmental aneuploidies and increases in resistance (MIC). Higher drug concentrations (supra-MIC) selected for increases in drug tolerance, but only rarely increased resistance. We then quantified the spectrum and effect of mutations after adaptation to the same four drug concentrations in a third genetic background that had a higher starting MIC (8 μg/ml). Strikingly, all lineages derived from this genetic background acquired trisomy of chromosome 5 and a concomitant increase in MIC during adaptation to the 8 μg/ml environment, but not during adaptation to lower or higher drug concentrations. These results identify that distinct drug response phenotypes are under selection at different drug concentrations and provide new evidence for consideration of initial MIC when treating challenging fungal infections.

## Results

### High Concentrations of Fluconazole Select for Drug Tolerance Phenotypes, low Concentrations Select for Resistance

To determine how *C. albicans* adapts to different physiologically relevant concentrations of FLC, we first compared two genetically distinct progenitors with the same initial MIC_50_ (SC5314 and P75063, MIC_50_ = 0.5 μg/ml FLC). Twelve single colonies from each progenitor were selected for parallel evolution experiments (SC5314: single colonies labeled A-L; P75063: single colonies labeled M-X), grown up in rich medium overnight and then split four ways into the following treatment lineages: 0 μg/ml FLC, 1 μg/ml FLC, 8 μg/ml FLC, and 64 μg/ml FLC (e.g., single colony A was divided into lineages A_0_, A_1_, A_8_, and A_64_). All 96 lineages were serially passaged every 72 h into fresh medium using a 1:1000 dilution. After 10 passages (∼100 generations) all 96 lineages underwent phenotypic analysis, and 48 lineages were selected for whole-genome sequencing prior to phenotypic analysis.

**Fig. 1.**
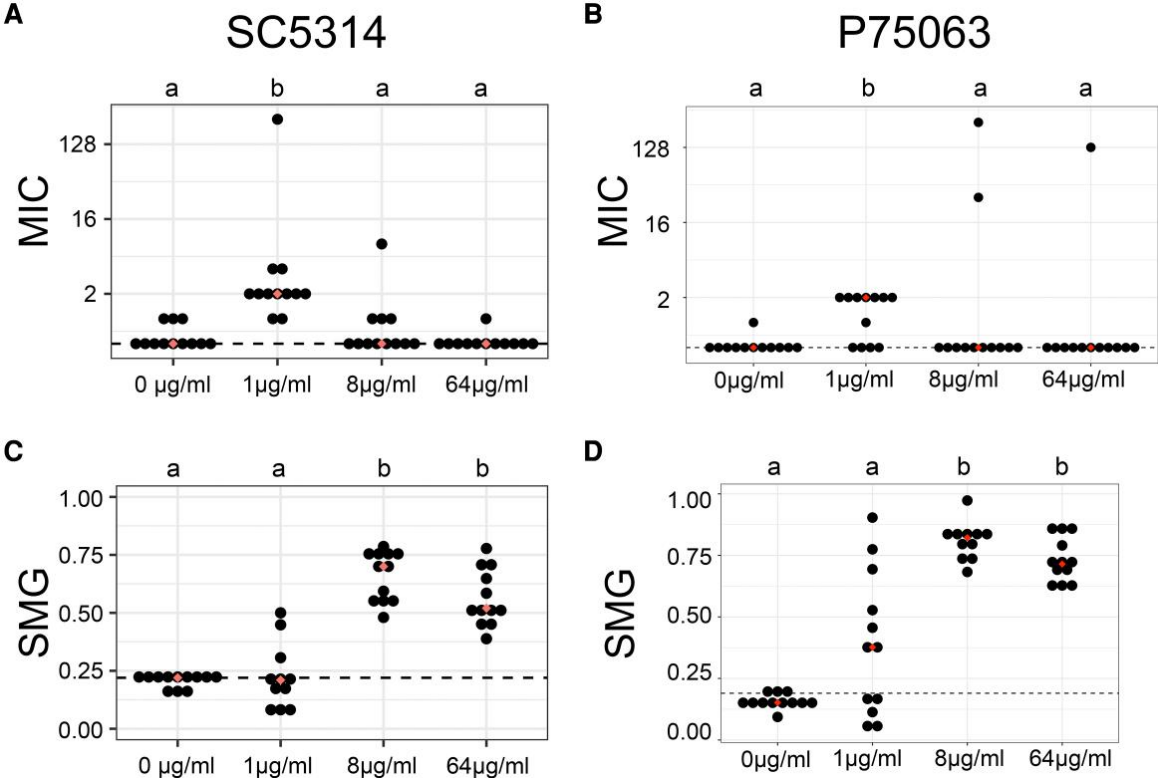
SC5314- and P75063-derived lineages exposed to 1 μg/ml FLC had a significant increase in MIC, whereas lineages grown in either 8 μg/ml or 64 μg/ml FLC had a significant increase in tolerance. MIC measured at 24 h for each of the 48 replicate lineages of (*A*) SC5314 and (*B*) P75063. Median MIC values for each treatment group (diamonds) and of the FLC-sensitive progenitors (dashed line) are indicated. Groups not sharing any letter are significantly different (*P* < 0.05, Kruskal-Wallis with Dunn's multiple comparison). Supra-MIC Growth values (SMG) measured at 48 h for each of the 48 replicate lineages of (*C*) SC5314 and (*D*) P75063. Median SMG values for each treatment group (diamonds) and of the FLC-sensitive progenitors (dashed line) are indicated. Groups not sharing any letter are significantly different (*P* < 0.05, Kruskal-Wallis with Dunn's multiple comparison). All MIC and SMG values represent three biological replicates.

We measured drug resistance (MIC_50_ at 24 h, abbreviated as MIC) and drug tolerance (Supra-MIC_50_ Growth, SMG, at 48 h) from all evolved lineages using a liquid microbroth dilution assay. MIC was quantified as the concentration of FLC that decreased the OD_600_ of the culture by ≥50% when compared with the no-drug control and SMG was calculated by taking the average OD_600_ value of the wells above the MIC_50_ at 48 h and dividing this average by the OD_600_ in the no-drug control well at 48 h (see Materials and Methods). All the FLC-evolved lineages acquired an increase in MIC and/or SMG relative to their progenitor (fig. 1 and Table 1; supplementary fig. S1, Supplementary Material online). The lineages evolved in 1 μg/ml FLC had a significant increase in MIC, in sharp contrast to lineages evolved in 0 μg/ml FLC, 8 μg/ml FLC, and 64 μg/ml FLC (fig. 1*A* and *B*, *P* < 0.05, Kruskal-Wallis with Dunn's multiple comparison). In contrast, lineages evolved in 8 and 64 μg/ml FLC tended to acquire significantly higher tolerance than lineages evolved in either 0 or 1 μg/ml FLC (fig. 1*C* and *D*, *P* < 0.05, Kruskal-Wallis with Dunn's multiple comparison). Resistance and tolerance levels were not significantly different between the lineages evolved in 8 and 64 μg/ml FLC, likely indicating that these two environments influenced the *C. albicans* populations similarly. These phenotypic results indicate that adaptation to different drug concentrations significantly influences the phenotypic trajectory of adaptation.

**Table 1.** Strains Used in This Study.

AMS Number	Description	Lineage Name	Progenitor	Evolution Experiment Drug Concentration (ug/ml FLC)	SRR	BioProject
2401	SC5314 Progenitor					
2794	P75063 Progenitor					
2885	FH1 Progenitor					
4040	SC5314 0ug FLC Passage 10 Colony 1	SC5314 A0	SC5314	0	SRR14929124	PRJNA741683
4041	SC5314 0ug FLC Passage 10 Colony 2	SC5314 B0	SC5314	0	SRR14929123	PRJNA741683
4042	SC5314 0ug FLC Passage 10 Colony 3	SC5314 C0	SC5314	0		
4043	SC5314 0ug FLC Passage 10 Colony 4	SC5314 D0	SC5314	0	SRR14929112	PRJNA741683
4044	SC5314 0ug FLC Passage 10 Colony 5	SC5314 E0	SC5314	0	SRR14929101	PRJNA741683
4045	SC5314 0ug FLC Passage 10 Colony 6	SC5314 F0	SC5314	0		
4046	SC5314 0ug FLC Passage 10 Colony 7	SC5314 G0	SC5314	0		
4047	SC5314 0ug FLC Passage 10 Colony 8	SC5314 H0	SC5314	0		
4048	SC5314 0ug FLC Passage 10 Colony 9	SC5314 I0	SC5314	0		
4049	SC5314 0ug FLC Passage 10 Colony 10	SC5314 J0	SC5314	0	SRR14929090	PRJNA741683
4050	SC5314 0ug FLC Passage 10 Colony 11	SC5314 K0	SC5314	0	SRR14929086	PRJNA741683
4051	SC5314 0ug FLC Passage 10 Colony 12	SC5314 L0	SC5314	0		
4052	SC5314 1ug FLC Passage 10 Colony 1	SC5314 A1	SC5314	1	SRR14929085	PRJNA741683
4053	SC5314 1ug FLC Passage 10 Colony 2	SC5314 B1	SC5314	1	SRR14929084	PRJNA741683
4054	SC5314 1ug FLC Passage 10 Colony 3	SC5314 C1	SC5314	1		
4055	SC5314 1ug FLC Passage 10 Colony 4	SC5314 D1	SC5314	1	SRR14929083	PRJNA741683
4056	SC5314 1ug FLC Passage 10 Colony 5	SC5314 E1	SC5314	1	SRR14929082	PRJNA741683
4057	SC5314 1ug FLC Passage 10 Colony 6	SC5314 F1	SC5314	1		
4058	SC5314 1ug FLC Passage 10 Colony 7	SC5314 G1	SC5314	1		
4059	SC5314 1ug FLC Passage 10 Colony 8	SC5314 H1	SC5314	1		
4060	SC5314 1ug FLC Passage 10 Colony 9	SC5314 I1	SC5314	1		
4061	SC5314 1ug FLC Passage 10 Colony 10	SC5314 J1	SC5314	1	SRR14929122	PRJNA741683
4062	SC5314 1ug FLC Passage 10 Colony 11	SC5314 K1	SC5314	1	SRR14929121	PRJNA741683
4063	SC5314 1ug FLC Passage 10 Colony 12	SC5314 L1	SC5314	1		
4064	SC5314 8ug FLC Passage 10 Colony 1	SC5314 A8	SC5314	8	SRR14929120	PRJNA741683
4065	SC5314 8ug FLC Passage 10 Colony 2	SC5314 B8	SC5314	8	SRR14929119	PRJNA741683
4066	SC5314 8ug FLC Passage 10 Colony 3	SC5314 C8	SC5314	8		
4067	SC5314 8ug FLC Passage 10 Colony 4	SC5314 D8	SC5314	8	SRR14929118	PRJNA741683
4068	SC5314 8ug FLC Passage 10 Colony 5	SC5314 E8	SC5314	8	SRR14929117	PRJNA741683
4069	SC5314 8ug FLC Passage 10 Colony 6	SC5314 F8	SC5314	8		
4070	SC5314 8ug FLC Passage 10 Colony 7	SC5314 G8	SC5314	8		
4071	SC5314 8ug FLC Passage 10 Colony 8	SC5314 H8	SC5314	8		
4072	SC5314 8ug FLC Passage 10 Colony 9	SC5314 I8	SC5314	8		
4073	SC5314 8ug FLC Passage 10 Colony 10	SC5314 J8	SC5314	8	SRR14929116	PRJNA741683
4074	SC5314 8ug FLC Passage 10 Colony 11	SC5314 K8	SC5314	8	SRR14929115	PRJNA741683
4075	SC5314 8ug FLC Passage 10 Colony 12	SC5314 L8	SC5314	8		
4076	SC5314 64 ug FLC Passage 10 Colony 1	SC5314 A64	SC5314	64	SRR14929114	PRJNA741683
4077	SC5314 64 ug FLC Passage 10 Colony 2	SC5314 B64	SC5314	64	SRR14929113	PRJNA741683
4078	SC5314 64 ug FLC Passage 10 Colony 3	SC5314 C64	SC5314	64		
4079	SC5314 64 ug FLC Passage 10 Colony 4	SC5314 D64	SC5314	64	SRR14929111	PRJNA741683
4080	SC5314 64 ug FLC Passage 10 Colony 5	SC5314 E64	SC5314	64	SRR14929110	PRJNA741683
4081	SC5314 64 ug FLC Passage 10 Colony 6	SC5314 F64	SC5314	64		
4082	SC5314 64 ug FLC Passage 10 Colony 7	SC5314 G64	SC5314	64		
4083	SC5314 64 ug FLC Passage 10 Colony 8	SC5314 H64	SC5314	64		
4084	SC5314 64 ug FLC Passage 10 Colony 9	SC5314 I64	SC5314	64		
4085	SC5314 64 ug FLC Passage 10 Colony 10	SC5314 J64	SC5314	64	SRR14929109	PRJNA741683
4086	SC5314 64 ug FLC Passage 10 Colony 11	SC5314 K64	SC5314	64	SRR14929108	PRJNA741683
4087	SC5314 64 ug FLC Passage 10 Colony 12	SC5314 L64	SC5314	64		
4088	P75063 0ug FLC Passage 10 Colony 1	P75063 M0	P75063	0		
4089	P75063 0ug FLC Passage 10 Colony 2	P75063 N0	P75063	0		
4090	P75063 0ug FLC Passage 10 Colony 3	P75063 O0	P75063	0		
4091	P75063 0ug FLC Passage 10 Colony 4	P75063 P0	P75063	0	SRR14929107	PRJNA741683
4092	P75063 0ug FLC Passage 10 Colony 5	P75063 Q0	P75063	0	SRR14929106	PRJNA741683
4093	P75063 0ug FLC Passage 10 Colony 6	P75063 R0	P75063	0	SRR14929105	PRJNA741683
4094	P75063 0ug FLC Passage 10 Colony 7	P75063 S0	P75063	0	SRR14929104	PRJNA741683
4095	P75063 0ug FLC Passage 10 Colony 8	P75063 T0	P75063	0	SRR14929103	PRJNA741683
4096	P75063 0ug FLC Passage 10 Colony 9	P75063 U0	P75063	0		
4097	P75063 0ug FLC Passage 10 Colony 10	P75063 V0	P75063	0		
4098	P75063 0ug FLC Passage 10 Colony 11	P75063 W0	P75063	0		
4099	P75063 0ug FLC Passage 10 Colony 12	P75063 X0	P75063	0	SRR14929102	PRJNA741683
4100	P75063 1ug FLC Passage 10 Colony 1	P75063 M1	P75063	1		
4101	P75063 1ug FLC Passage 10 Colony 2	P75063 N1	P75063	1		
4102	P75063 1ug FLC Passage 10 Colony 3	P75063 O1	P75063	1		
4103	P75063 1ug FLC Passage 10 Colony 4	P75063 P1	P75063	1	SRR14929100	PRJNA741683
4104	P75063 1ug FLC Passage 10 Colony 5	P75063 Q1	P75063	1	SRR11347410	PRJNA613282
4105	P75063 1ug FLC Passage 10 Colony 6	P75063 R1	P75063	1	SRR11347409	PRJNA613282
4106	P75063 1ug FLC Passage 10 Colony 7	P75063 S1	P75063	1	SRR11347408	PRJNA613282
4107	P75063 1ug FLC Passage 10 Colony 8	P75063 T1	P75063	1	SRR11347407	PRJNA613282
4108	P75063 1ug FLC Passage 10 Colony 9	P75063 U1	P75063	1		
4109	P75063 1ug FLC Passage 10 Colony 10	P75063 V1	P75063	1		
4110	P75063 1ug FLC Passage 10 Colony 11	P75063 W1	P75063	1		
4111	P75063 1ug FLC Passage 10 Colony 12	P75063 X1	P75063	1	SRR8324566	PRJNA510147
4112	P75063 8ug FLC Passage 10 Colony 1	P75063 M8	P75063	8	SRR14996374	PRJNA741683
4113	P75063 8ug FLC Passage 10 Colony 2	P75063 N8	P75063	8		
4114	P75063 8ug FLC Passage 10 Colony 3	P75063 O8	P75063	8		
4115	P75063 8ug FLC Passage 10 Colony 4	P75063 P8	P75063	8	SRR14929099	PRJNA741683
4116	P75063 8ug FLC Passage 10 Colony 5	P75063 Q8	P75063	8	SRR14929098	PRJNA741683
4117	P75063 8ug FLC Passage 10 Colony 6	P75063 R8	P75063	8	SRR14929097	PRJNA741683
4118	P75063 8ug FLC Passage 10 Colony 7	P75063 S8	P75063	8	SRR14929096	PRJNA741683
4119	P75063 8ug FLC Passage 10 Colony 8	P75063 T8	P75063	8	SRR14929095	PRJNA741683
4120	P75063 8ug FLC Passage 10 Colony 9	P75063 U8	P75063	8		
4121	P75063 8ug FLC Passage 10 Colony 10	P75063 V8	P75063	8		
4122	P75063 8ug FLC Passage 10 Colony 11	P75063 W8	P75063	8		
4123	P75063 8ug FLC Passage 10 Colony 12	P75063 X8	P75063	8	SRR14929094	PRJNA741683
4124	P75063 64ug FLC Passage 10 Colony 1	P75063 M64	P75063	64	SRR14996373	PRJNA741683
4125	P75063 64ug FLC Passage 10 Colony 2	P75063 N64	P75063	64		
4126	P75063 64ug FLC Passage 10 Colony 3	P75063 O64	P75063	64		
4127	P75063 64ug FLC Passage 10 Colony 4	P75063 P64	P75063	64	SRR14929093	PRJNA741683
4128	P75063 64ug FLC Passage 10 Colony 5	P75063 Q64	P75063	64	SRR14929092	PRJNA741683
4129	P75063 64ug FLC Passage 10 Colony 6	P75063 R64	P75063	64	SRR14929091	PRJNA741683
4130	P75063 64ug FLC Passage 10 Colony 7	P75063 S64	P75063	64	SRR14929089	PRJNA741683
4131	P75063 64ug FLC Passage 10 Colony 8	P75063 T64	P75063	64	SRR14929088	PRJNA741683
4132	P75063 64ug FLC Passage 10 Colony 9	P75063 U64	P75063	64		
4133	P75063 64ug FLC Passage 10 Colony 10	P75063 V64	P75063	64		
4134	P75063 64ug FLC Passage 10 Colony 11	P75063 W64	P75063	64		
4135	P75063 64ug FLC Passage 10 Colony 12	P75063 X64	P75063	64	SRR14929087	PRJNA741683
4184	FH1 0ug FLC Passage 10 Colony 1	FH1-A0	FH1	0	SRR21225682	PRJNA741683
4185	FH1 0ug FLC Passage 10 Colony 2	FH1-B0	FH1	0	SRR21225681	PRJNA741683
4186	FH1 0ug FLC Passage 10 Colony 3	FH1-C0	FH1	0	SRR21225670	PRJNA741683
4187	FH1 0ug FLC Passage 10 Colony 4	FH1-D0	FH1	0	SRR21225659	PRJNA741683
4188	FH1 0ug FLC Passage 10 Colony 5	FH1-E0	FH1	0	SRR21225648	PRJNA741683
4189	FH1 0ug FLC Passage 10 Colony 6	FH1-F0	FH1	0	SRR21225639	PRJNA741683
4190	FH1 0ug FLC Passage 10 Colony 7	FH1-G0	FH1	0	SRR21225638	PRJNA741683
4191	FH1 0ug FLC Passage 10 Colony 8	FH1-H0	FH1	0	SRR21225637	PRJNA741683
4192	FH1 0ug FLC Passage 10 Colony 9	FH1-I0	FH1	0	SRR21225636	PRJNA741683
4193	FH1 0ug FLC Passage 10 Colony 10	FH1-J0	FH1	0	SRR21225635	PRJNA741683
4194	FH1 0ug FLC Passage 10 Colony 11	FH1-K0	FH1	0	SRR21225680	PRJNA741683
4195	FH1 0ug FLC Passage 10 Colony 12	FH1-L0	FH1	0	SRR21225679	PRJNA741683
4196	FH1 1ug FLC Passage 10 Colony 1	FH1-A1	FH1	1	SRR21225678	PRJNA741683
4197	FH1 1ug FLC Passage 10 Colony 2	FH1-B1	FH1	1	SRR21225677	PRJNA741683
4198	FH1 1ug FLC Passage 10 Colony 3	FH1-C1	FH1	1	SRR21225676	PRJNA741683
4199	FH1 1ug FLC Passage 10 Colony 4	FH1-D1	FH1	1	SRR21225675	PRJNA741683
4200	FH1 1ug FLC Passage 10 Colony 5	FH1-E1	FH1	1	SRR21225674	PRJNA741683
4201	FH1 1ug FLC Passage 10 Colony 6	FH1-F1	FH1	1	SRR21225673	PRJNA741683
4202	FH1 1ug FLC Passage 10 Colony 7	FH1-G1	FH1	1	SRR21225672	PRJNA741683
4203	FH1 1ug FLC Passage 10 Colony 8	FH1-H1	FH1	1	SRR21225671	PRJNA741683
4204	FH1 1ug FLC Passage 10 Colony 9	FH1-I1	FH1	1	SRR21225669	PRJNA741683
4205	FH1 1ug FLC Passage 10 Colony 10	FH1-J1	FH1	1	SRR21225668	PRJNA741683
4206	FH1 1ug FLC Passage 10 Colony 11	FH1-K1	FH1	1	SRR21225667	PRJNA741683
4207	FH1 1ug FLC Passage 10 Colony 12	FH1-L1	FH1	1	SRR21225666	PRJNA741683
4208	FH1 8ug FLC Passage 10 Colony 1	FH1-A8	FH1	8	SRR21225665	PRJNA741683
4209	FH1 8ug FLC Passage 10 Colony 2	FH1-B8	FH1	8	SRR21225664	PRJNA741683
4210	FH1 8ug FLC Passage 10 Colony 3	FH1-C8	FH1	8	SRR21225663	PRJNA741683
4211	FH1 8ug FLC Passage 10 Colony 4	FH1-D8	FH1	8	SRR21225662	PRJNA741683
4212	FH1 8ug FLC Passage 10 Colony 5	FH1-E8	FH1	8	SRR21225661	PRJNA741683
4213	FH1 8ug FLC Passage 10 Colony 6	FH1-F8	FH1	8	SRR21225660	PRJNA741683
4214	FH1 8ug FLC Passage 10 Colony 7	FH1-G8	FH1	8	SRR21225658	PRJNA741683
4215	FH1 8ug FLC Passage 10 Colony 8	FH1-H8	FH1	8	SRR21225657	PRJNA741683
4216	FH1 8ug FLC Passage 10 Colony 9	FH1-I8	FH1	8	SRR21225656	PRJNA741683
4217	FH1 8ug FLC Passage 10 Colony 10	FH1-J8	FH1	8	SRR21225655	PRJNA741683
4218	FH1 8ug FLC Passage 10 Colony 11	FH1-K8	FH1	8	SRR21225654	PRJNA741683
4219	FH1 8ug FLC Passage 10 Colony 12	FH1-L8	FH1	8	SRR21225653	PRJNA741683
4220	FH1 64 ug FLC Passage 10 Colony 1	FH1-A64	FH1	64	SRR21225652	PRJNA741683
4221	FH1 64 ug FLC Passage 10 Colony 2	FH1-B64	FH1	64	SRR21225651	PRJNA741683
4222	FH1 64 ug FLC Passage 10 Colony 3	FH1-C64	FH1	64	SRR21225650	PRJNA741683
4223	FH1 64 ug FLC Passage 10 Colony 4	FH1-D64	FH1	64	SRR21225649	PRJNA741683
4224	FH1 64 ug FLC Passage 10 Colony 5	FH1-E64	FH1	64	SRR21225647	PRJNA741683
4225	FH1 64 ug FLC Passage 10 Colony 6	FH1-F64	FH1	64	SRR21225646	PRJNA741683
4226	FH1 64 ug FLC Passage 10 Colony 7	FH1-G64	FH1	64	SRR21225645	PRJNA741683
4227	FH1 64 ug FLC Passage 10 Colony 8	FH1-H64	FH1	64	SRR21225644	PRJNA741683
4228	FH1 64 ug FLC Passage 10 Colony 9	FH1-I64	FH1	64	SRR21225643	PRJNA741683
4229	FH1 64 ug FLC Passage 10 Colony 10	FH1-J64	FH1	64	SRR21225642	PRJNA741683
4230	FH1 64 ug FLC Passage 10 Colony 11	FH1-K64	FH1	64	SRR21225641	PRJNA741683
4231	FH1 64 ug FLC Passage 10 Colony 12	FH1-L64	FH1	64	SRR21225640	PRJNA741683

### Frequent Polyploidization Events are Selected for During Adaptation to Fluconazole

**Fig. 2.**
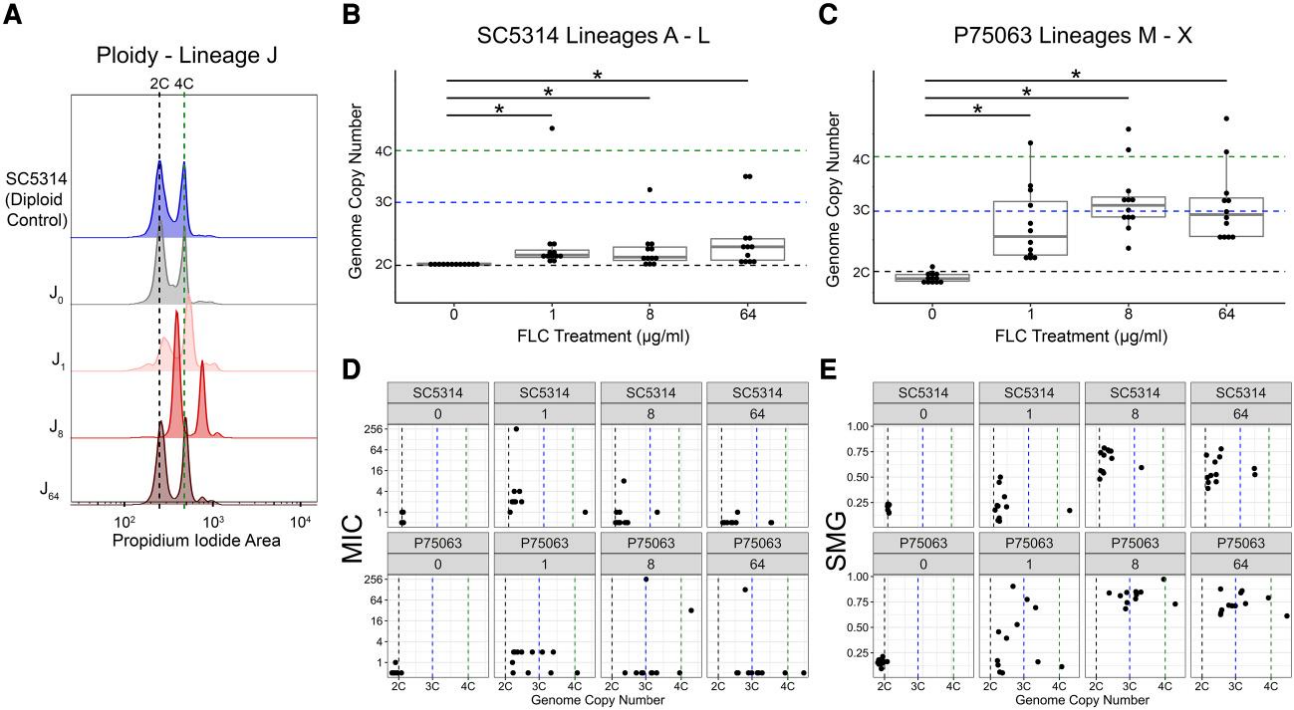
Genome copy number increase during prolonged fluconazole exposure. (*A*) Representative ploidy analysis of lineage J derived from P75063. Dashed lines indicate the 2*C* and 4*C* genome copy number. Genome copy number plotted as fluorescence intensity of propidium iodide-labeled cells for the 48 replicate lineages from (*B*) SC5314 and (*C*) P75063 (see Materials and Methods). Median genome copy number was estimated as the first (G1) propidium iodide (PI) peak that contained >10% of the total population of cells. Dashed horizontal lines indicate the 2*C*, 3*C*, and 4*C* genome copy numbers based off the SC5314 and P75063 progenitor genome size. There is a significant increase in median genome size for the FLC-evolved lineages from SC5314 (*P* < 0.05, Kruskal-Wallis with Dunn's multiple comparison) and P75063 (*P* < 0.05, Kruskal-Wallis with Dunn's multiple comparison). There is a significant increase in median genome size between lineages derived from SC5314 and P75063 for the 8 μg/ml treatment group (*P* < 0.01, Mann–Whitney *U* test) and for the 64 μg/ml treatment group (*P* < 0.01, Mann–Whitney *U* test). Asterisk above graph indicates significant differences compared with the 0 μg/ml treatment group (*P* < 0.05 Kruskal-Wallis with Dunn's multiple comparison). (*D*) MIC and (*E*) SMG measurements from figure 1 by genome copy number, faceted by each progenitor and evolution treatment. Median genome copy numbers (2*C*, 3*C*, 4*C*) are the same as above. Lineages with an MIC >256 μg/ml FLC were excluded from the SMG plot. Linear regressions for each panel were conducted, with no significant correlations (Table 2, Tab 2).

To determine what genotypic changes underlie the phenotypic differences at each drug concentration, we performed comprehensive comparative genomics using flow cytometry and whole-genome sequencing. Flow cytometry quantifies total DNA content by measuring fluorescence after staining with propidium iodide. Lineages with a range of both ploidy and aneuploidy levels are detected by an increase or decrease in fluorescence relative to the diploid progenitor strain (fig. 2*A*) (Todd et al. 2018). All 0 μg/ml FLC evolved lineages remained diploid (fig. 2*B* and *C*, Table 2), whereas FLC-evolved lineages exhibited a significant increase in genome size (*P* < 0.05, Kruskal-Wallis with Dunn's multiple comparison). These results indicate that karyotypic changes occurred at all three drug concentrations, though the degree of genome size increase was influenced by strain background.

**Table 2.** MIC, SMG, Ploidy (Tab 1), Linear Regressions (Tab 2).

Strain Number	Lineage Name	Progenitor	MIC50 (24 h)	SMG (48 h)	Ploidy Peak 1 (AU)	Ploidy Peak 2 (AU)	Estimated Base Ploidy
2401	SC5314	Progenitor	0.5	0.23	251	468	2N
2794	P75063	Progenitor	0.5	0.14	240	457	2N
2885	FH1	Progenitor	8.00	0.14	275	506	2N
4040	A0	SC5314	0.50	0.23	251	474	2N
4041	B0	SC5314	1.00	0.18	251	473	2N
4042	C0	SC5314	0.50	0.23	255	481	2N
4043	D0	SC5314	1.00	0.15	255	482	2N
4044	E0	SC5314	0.50	0.21	250	475	2N
4045	F0	SC5314	0.50	0.21	251	469	2N
4046	G0	SC5314	1.00	0.15	255	478	2N
4047	H0	SC5314	0.50	0.23	253	478	2N
4048	I0	SC5314	0.50	0.21	251	477	2N
4049	J0	SC5314	0.50	0.23	256	476	2N
4050	K0	SC5314	0.50	0.23	253	478	2N
4051	L0	SC5314	0.50	0.22	254	479	2N
4052	A1	SC5314	2.00	0.22	263	490	2N
4053	B1	SC5314	2.00	0.07	266	503	2N
4054	C1	SC5314	2.00	0.21	295	545	2N
4055	D1	SC5314	2.00	0.22	266	494	2N
4056	E1	SC5314	2.00	0.10	270	498	2N
4057	F1	SC5314	1.00	0.17	510	1003	4N
4058	G1	SC5314	256.00	NA	277	509	2N
4059	H1	SC5314	4.00	0.45	270	503	2N
4060	I1	SC5314	1.00	0.18	256	496	2N
4061	J1	SC5314	4.00	0.31	288	538	2N
4062	K1	SC5314	2.00	0.07	271	501	2N
4063	L1	SC5314	2.00	0.50	274	502	2N
4064	A8	SC5314	0.50	0.55	262	512	2N
4065	B8	SC5314	1.00	0.72	266	508	2N
4066	C8	SC5314	1.00	0.56	255	487	2N
4067	D8	SC5314	0.50	0.68	293	530	2N
4068	E8	SC5314	0.50	0.76	284	534	2N
4069	F8	SC5314	8.00	0.77	280	544	2N
4070	G8	SC5314	0.50	0.79	267	501	2N
4071	H8	SC5314	0.50	0.54	265	516	2N
4072	I8	SC5314	0.50	0.76	290	555	2N
4073	J8	SC5314	1.00	0.59	394	766	3N
4074	K8	SC5314	0.50	0.74	255	493	2N
4075	L8	SC5314	0.50	0.48	252	490	2N
4076	A64	SC5314	0.50	0.65	284	521	2N
4077	B64	SC5314	0.50	0.70	300	551	2N
4078	C64	SC5314	0.50	0.72	255	489	2N
4079	D64	SC5314	0.50	0.52	420	801	3N
4080	E64	SC5314	1.00	0.78	305	577	2N
4081	F64	SC5314	0.50	0.50	258	501	2N
4082	G64	SC5314	0.50	0.45	288	545	2N
4083	H64	SC5314	0.50	0.39	260	495	2N
4084	I64	SC5314	0.50	0.58	418	792	3N
4085	J64	SC5314	0.50	0.45	261	494	2N
4086	K64	SC5314	0.50	0.52	270	513	2N
4087	L64	SC5314	0.50	0.53	288	540	2N
4088	M0	P75063	0.50	0.16	249	464	2N
4089	N0	P75063	0.50	0.16	236	443	2N
4090	O0	P75063	0.50	0.14	225	427	2N
4091	P0	P75063	1.00	0.09	229	438	2N
4092	Q0	P75063	0.50	0.15	217	418	2N
4093	R0	P75063	0.50	0.14	222	428	2N
4094	S0	P75063	0.50	0.18	223	426	2N
4095	T0	P75063	0.50	0.18	220	423	2N
4096	U0	P75063	0.50	0.14	221	422	2N
4097	V0	P75063	0.50	0.21	234	441	2N
4098	W0	P75063	0.50	0.15	235	445	2N
4099	X0	P75063	0.50	0.14	228	430	2N
4100	M1	P75063	2.00	0.16	402	686	3N
4101	N1	P75063	0.50	0.11	483	919	4N
4102	O1	P75063	2.00	0.05	281	514	2N
4103	P1	P75063	1.00	0.17	264	505	2N
4104	Q1	P75063	2.00	0.06	272	505	2N
4105	R1	P75063	2.00	0.53	331	617	2N
4106	S1	P75063	2.00	0.46	268	507	2N
4107	T1	P75063	0.50	0.13	266	535	2N
4108	U1	P75063	0.50	0.69	394	751	3N
4109	V1	P75063	2.00	0.77	365	695	3N
4110	W1	P75063	0.50	0.90	317	629	2N
4111	X1	P75063	2.00	0.39	295	566	2N
4112	M8	P75063	32.00	0.73	509	956	4N
4113	N8	P75063	256.00	NA	356	632	3N
4114	O8	P75063	0.50	0.97	470	934	4N
4115	P8	P75063	0.50	0.78	374	659	3N
4116	Q8	P75063	0.50	0.74	345	638	2N
4117	R8	P75063	0.50	0.84	344	606	2N
4118	S8	P75063	0.50	0.82	377	648	3N
4119	T8	P75063	0.50	0.85	392	661	3N
4120	U8	P75063	0.50	0.84	284	541	2N
4121	V8	P75063	0.50	0.85	375	636	3N
4122	W8	P75063	0.50	0.68	340	631	2N
4123	X8	P75063	0.50	0.81	322	596	2N
4124	M64	P75063	128.00	0.72	331	596	2N
4125	N64	P75063	0.50	0.79	466	881	4N
4126	O64	P75063	0.50	0.73	388	748	3N
4127	P64	P75063	0.50	0.84	372	648	3N
4128	Q64	P75063	0.50	0.71	342	554	2N
4129	R64	P75063	0.50	0.67	307	585	2N
4130	S64	P75063	0.50	0.88	303	589	2N
4131	T64	P75063	0.50	0.86	376	648	3N
4132	U64	P75063	0.50	0.71	353	612	2N
4133	V64	P75063	0.50	0.61	529	1017	4N
4134	W64	P75063	0.50	0.64	304	574	2N
4135	X64	P75063	0.50	0.63	303	565	2N
4184	FH1-A0	FH1	8.00	0.16	273	495	2N
4185	FH1-B0	FH1	8.00	0.12	275	499	2N
4186	FH1-C0	FH1	8.00	0.13	275	498	2N
4187	FH1-D0	FH1	8.00	0.15	271	496	2N
4188	FH1-E0	FH1	8.00	0.12	273	498	2N
4189	FH1-F0	FH1	8.00	0.13	273	500	2N
4190	FH1-G0	FH1	8.00	0.16	275	500	2N
4191	FH1-H0	FH1	8.00	0.16	265	483	2N
4192	FH1-I0	FH1	8.00	0.20	273	499	2N
4193	FH1-J0	FH1	8.00	0.16	271	493	2N
4194	FH1-K0	FH1	8.00	0.18	265	492	2N
4195	FH1-L0	FH1	8.00	0.16	268	487	2N
4196	FH1-A1	FH1	8.00	0.13	259	476	2N
4197	FH1-B1	FH1	8.00	0.15	268	490	2N
4198	FH1-C1	FH1	8.00	0.13	271	500	2N
4199	FH1-D1	FH1	8.00	0.14	272	494	2N
4200	FH1-E1	FH1	8.00	0.15	273	496	2N
4201	FH1-F1	FH1	8.00	0.18	273	497	2N
4202	FH1-G1	FH1	8.00	0.19	272	498	2N
4203	FH1-H1	FH1	8.00	0.16	273	501	2N
4204	FH1-I1	FH1	8.00	0.16	271	498	2N
4205	FH1-J1	FH1	8.00	0.16	270	492	2N
4206	FH1-K1	FH1	8.00	0.19	274	496	2N
4207	FH1-L1	FH1	8.00	0.16	269	490	2N
4208	FH1-A8	FH1	16.00	0.22	281	509	2N
4209	FH1-B8	FH1	16.00	0.20	283	512	2N
4210	FH1-C8	FH1	16.00	0.21	283	515	2N
4211	FH1-D8	FH1	16.00	0.21	280	516	2N
4212	FH1-E8	FH1	16.00	0.21	282	515	2N
4213	FH1-F8	FH1	16.00	0.25	284	519	2N
4214	FH1-G8	FH1	16.00	0.13	284	515	2N
4215	FH1-H8	FH1	16.00	0.19	278	506	2N
4216	FH1-I8	FH1	16.00	0.14	282	513	2N
4217	FH1-J8	FH1	16.00	0.16	276	504	2N
4218	FH1-K8	FH1	16.00	0.18	275	501	2N
4219	FH1-L8	FH1	16.00	0.16	278	512	2N
4220	FH1-A64	FH1	8.00	0.20	260	476	2N
4221	FH1-B64	FH1	8.00	0.13	255	468	2N
4222	FH1-C64	FH1	8.00	0.19	261	474	2N
4223	FH1-D64	FH1	8.00	0.23	254	470	2N
4224	FH1-E64	FH1	8.00	0.15	266	477	2N
4225	FH1-F64	FH1	8.00	0.22	255	473	2N
4226	FH1-G64	FH1	8.00	0.16	251	466	2N
4227	FH1-H64	FH1	8.00	0.21	251	465	2N
4228	FH1-I64	FH1	8.00	0.41	249	462	2N
4229	FH1-J64	FH1	8.00	0.24	252	464	2N
4230	FH1-K64	FH1	8.00	0.22	255	476	2N
4231	FH1-L64	FH1	8.00	0.17	257	474	2N

Dependent Variable	Lineage	Treatment	r^2^	*P*-value
MIC	SC5314	0	0.5386979	0.4940819
MIC	SC5314	1	0.6555458	0.8215157
MIC	SC5314	8	0.8998948	0.9041320
MIC	SC5314	64	0.0553069	0.9407856
MIC	P75063	0	0.7077209	0.9355935
MIC	P75063	1	0.0503718	0.3794936
MIC	P75063	8	0.8509903	0.9828895
MIC	P75063	64	0.5270764	0.6416587
SMG	SC5314	0	0.4033163	0.5023129
SMG	SC5314	1	0.1859859	0.7814282
SMG	SC5314	8	0.0272821	0.9589428
SMG	SC5314	64	0.0326302	0.7443201
SMG	P75063	0	0.8415620	0.6551474
SMG	P75063	1	0.6858302	0.6905251
SMG	P75063	8	0.0008318	0.6298133
SMG	P75063	64	0.0005182	0.8647029

Linear regression models were individually fitted to predict MIC from PI Peak 1 (see MIC, SMG, Ploidy tab) for each Lineage (SC5314, P75063) and Treatment (0, 1, 8, 64 µg/ml FLC) group. This was repeated for SMG. The r^2^ value and the *P*-value from each linear regression are reported above.

To determine if polyploidization (i.e., evolved a base ploidy of ∼3N or 4N) correlated with resistance or tolerance phenotypes, we compared the median genome size with the MIC and SMG values from figure 1. We found that there was no significant correlation between increasing genome size and MIC or SMG in either strain background for any drug concentration (fig. 2*D* and *E*, Linear regressions Table 2-Tab 2). We performed growth curve analysis on the lineages with the highest ploidy levels to better understand how they reached fixation. All lineages tested had significantly improved growth in 1 μg/ml FLC compared with the diploid progenitors, and in the absence of drug only some SC5314-derived polyploid lineages had a small fitness cost (supplementary fig. S2, Supplementary Material online). In summary, all evolved lineages have increased their ability to respond to drug in the presence of FLC, but the degree of genome size increase is not differentially driving increases in MIC and SMG.

### Segmental Aneuploidies are Fluconazole Concentration-dependent

**Fig. 3.**
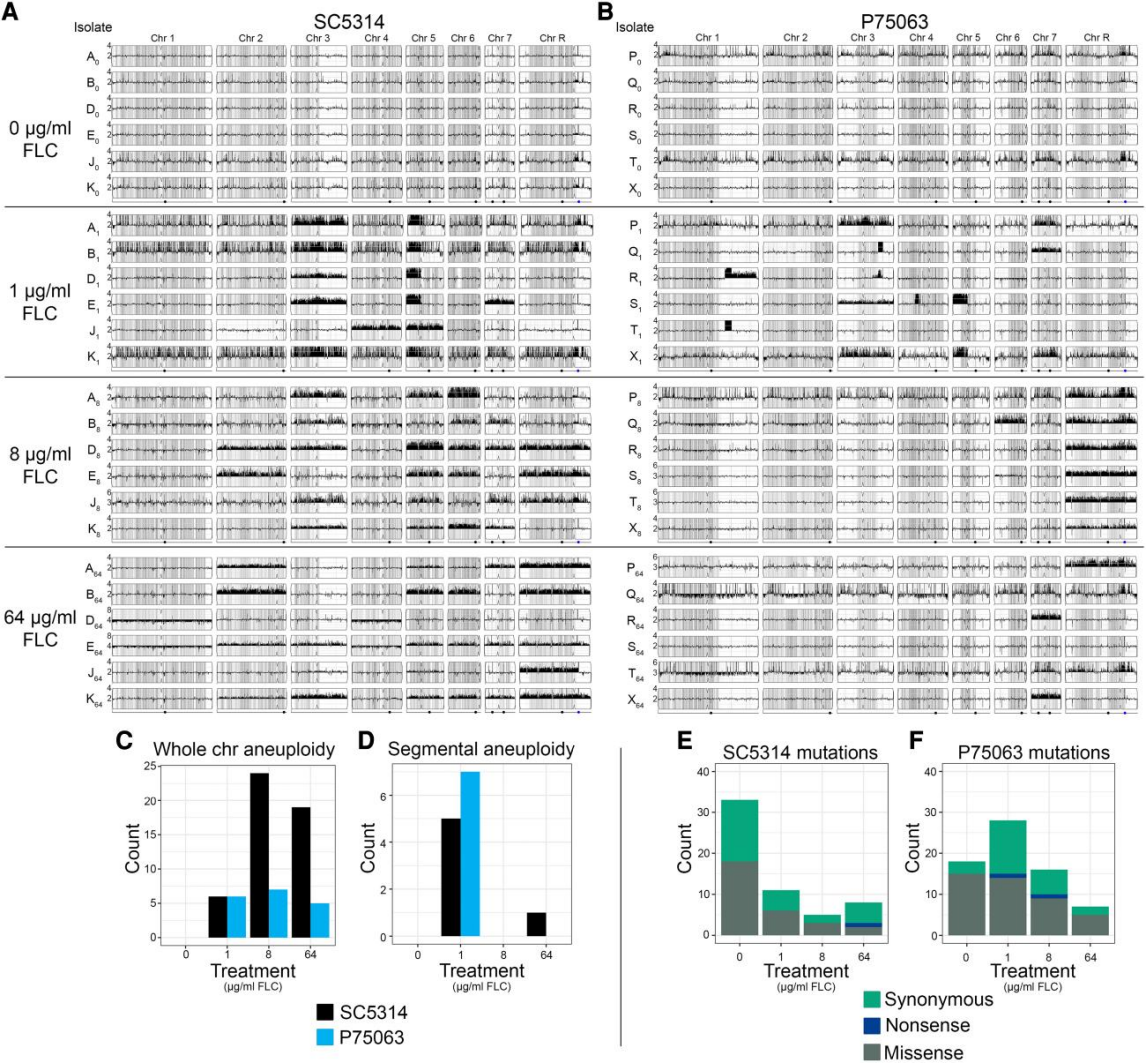
Fluconazole concentration impacts the mutational spectrum of evolved lineages. Whole-genome sequence data plotted as the log2 ratio and converted to chromosome copy number (y-axis, 1–8 copies) as a function of chromosome position (x-axis, Chr1-ChrR) using the Yeast Mapping Analysis Pipeline (YMAP; [Abbey et al. 2014]). The baseline chromosome copy number (ploidy) was determined by flow cytometry (see Materials and Methods, and fig. 2). Lineages of (*A*) SC5314 and (*B*) P75063 are grouped by drug treatment (0 μg/ml FLC, 1 μg/ml FLC, 8 μg/ml FLC, and 64 μg/ml FLC). Gray shading indicates heterozygous positions throughout the genome, which are distinct between the two progenitors, with darker gray regions containing more heterozygous loci and white regions containing few or no heterozygous loci. Centromere position indicated by a notch on every chromosome. Dots below the bottom YMAP of each drug treatment group identifies the location of the Major Repeat Sequences (MRS) and the ribosomal DNA array (rDNA). Histogram of (C) whole-chromosome aneuploidy events and (*D*) segmental aneuploidy events detected by read depth analysis of lineages derived from SC5314 (black bars) and P75063 (blue bars). Segmental aneuploidies predominantly occur at 1 μg/ml FLC for lineages derived from both progenitor isolates. Frequency and type of de novo single-nucleotide variants identified in the 48 replicate lineages of (*E*) SC5314 and (*F*) P75063.

To determine the full spectrum of mutations that arose during the evolution experiment and to provide additional mechanistic insight into adaptation beyond ploidy changes, populations evolved in all four drug concentrations from six lineages from each progenitor (e.g., A_0_, A_1_, A_8_, A_64_; B_0_, B_1_, B_8_, B_64_, etc.) were selected for whole-genome sequencing (48 lineages in total). All 12 of the lineages evolved in 0 μg/ml FLC remained euploid by chromosome copy number analysis. In contrast, aneuploidy was detected in 35/36 of the FLC-evolved lineages (fig. 3*A* and *B*; only lineage S_64_ remained euploid diploid). Amplification of Chromosome R, containing the ribosomal DNA array, was the most common aneuploidy identified at both 8 and 64 μg/ml FLC. Most lineages contained multiple aneuploid chromosomes, and lineages derived from SC5314 had nearly four times the number of whole-chromosome aneuploidy events than the lineages derived from P75063 at both 8 and 64 μg/ml FLC (fig. 3*C*). This suggests that ploidy analysis by flow cytometry may be underestimating the frequency of chromosome copy number changes, that are more common than polyploidization, in the SC5314 background.

Segmental aneuploidies that amplified only a portion of a chromosome occurred almost exclusively in the lineages evolved at 1 μg/ml FLC (fig. 3*D*, 5/6 lineages from both progenitors). Amplification of the left arm of Chromosome 5 in an isochromosome structure (i(5L)) was the most frequent segmental aneuploidy in both genetic backgrounds (SC5314: 5/6 lineages, P75063: 2/6 lineages). This is the first report of i(5L) formation in the reference strain SC5314 and the first indication that drug concentration can impact selection for, and possibly formation of, this recurrent segmental aneuploidy. Five additional segmental aneuploidies of Chromosomes 1, 3, and 4 amplified from 3 to 13 copies per genome in P75063 lineages evolved in 1 μg/ml FLC (fig. 3*B*; supplementary figs. S3 and S4, Supplementary Material online). The copy number breakpoints of all segmental aneuploidies occurred at long repeat sequences as described previously (Todd et al. 2019; Todd and Selmecki 2020). Only one segmental aneuploidy was identified above 1 μg/ml FLC (Lineage J_64_) and occurred at the rDNA array on ChrR, a common copy number breakpoint in clinical isolates. The general lack of segmental aneuploidies observed at 8 and 64 μg/ml is surprising, especially given the frequency of whole-chromosome aneuploid events observed at these drug concentrations.

We then quantified LOH events across all lineages. Ten LOH events were detected in seven of the 48 lineages, including whole-chromosome LOH events (5/10) and segmental chromosome LOH events (5/10) ranging from ∼8 kb to ∼716 kb (Table 3). LOH events occurred almost exclusively in the lineages evolved at 1 μg/ml FLC (9/10 LOH events). Most of the LOH events occurred in SC5314 (7/10) compared with P75063 (3/10). The segmental chromosome LOH events frequently occurred at long inverted repeat sequences similar to the copy number breakpoints previously implicated in FLC adaptation (Todd et al. 2019). In two different SC5314 lineages, B_1_ and D_1_, LOH of all or part of the right arm of Chr5 was associated with amplification of the left arm of Chr5 in an i(5L) isochromosome. Therefore, the inverted repeat sequence flanking the centromere of Chr5 was involved in both copy number variation and LOH (Selmecki et al. 2009). Additionally, whole-ChrR LOH was found in both progenitors at 1 μg/ml, SC5314 J_1_ and P75063 P_1_.

**Table 3.** Single-Nucleotide Variants (Tab 1) and Loss of Heterozygosity Events (Tab 2).

Strain Number	Lineage Name	Treatment	Progenitor	Mutation Type	Chromosome	Position	Reference Allele	Alternate Allele	Parental Reference Freq.	Parent Alternate Freq.	Evolved Reference Freq.	Evolved Alternate Freq.	Amino Acid Change	Gene ID	Systematic ID	Gene ID (if exists)	Gene Description
4040	A0	0 ug/mL FLC	SC5314	Synonymous	1	1775675	T	A	1	0	0.88	0.1		orf19.5089	C1_08130C_A	TERT	Telomerase reverse transcriptase; catalytic protein subunit of telomere synthesis; essential for telomerase activity; has telomerase-specific motif T and other conserved reverse transcriptase motifs
4040	A0	0 ug/mL FLC	SC5314	Synonymous	1	1353027	C	T	1	0	0.91	0.07		orf19.6276	C1_06350W_A		Protein of unknown function; rat catheter biofilm repressed
4041	B0	0 ug/mL FLC	SC5314	Synonymous	7	764042	T	C	1	0	0.8	0.2		orf19.1330	C7_03460W_A		Protein of unknown function
4041	B0	0 ug/mL FLC	SC5314	Synonymous	4	897645	T	C	1	0	0.84	0.16		orf19.5290	C4_04200C_A		Protein of unknown function; repressed by Sfu1; Hap43-induced gene
4041	B0	0 ug/mL FLC	SC5314	Synonymous	1	1775657	T	A	1	0	0.86	0.14		orf19.5089	C1_08130C_A	TERT	Telomerase reverse transcriptase; catalytic protein subunit of telomere synthesis; essential for telomerase activity; has telomerase-specific motif T and other conserved reverse transcriptase motifs
4041	B0	0 ug/mL FLC	SC5314	Missense	3	801887	A	C	1	0	0.73	0.27	Asn611His	orf19.6936	C3_03810W_A	RAD53	Protein involved in regulation of DNA-damage-induced filamentous growth; putative component of cell cycle checkpoint; ortholog of S. cerevisiae Rad53p, protein kinase required for cell-cycle arrest in response to DNA damage
4041	B0	0 ug/mL FLC	SC5314	Missense	1	1775655	C	T	1	0	0.86	0.14	Ser602Asn	orf19.5089	C1_08130C_A	TERT	Telomerase reverse transcriptase; catalytic protein subunit of telomere synthesis; essential for telomerase activity; has telomerase-specific motif T and other conserved reverse transcriptase motifs
4041	B0	0 ug/mL FLC	SC5314	Missense	R	2020623	T	C	1	0	0.91	0.09	Ser1107Pro	orf19.7342	CR_09470W_A	AXL1	Putative endoprotease; induced by alpha factor; transcript is upregulated in an RHE model of oral candidiasis and in clinical isolates from HIV + patients with oral candidiasis
4043	D0	0 ug/mL FLC	SC5314	Synonymous	3	664300	A	G	1	0	0.67	0.33		orf19.309	C3_03120C_A	DAL5	Allantoate permease; nitrogen catabolite repressed, induced in absence of preferred N sources; nitrogen source regulation requires Gat1; possibly essential gene (by UAU1 method); Hap43-repressed
4043	D0	0 ug/mL FLC	SC5314	Synonymous	2	706855	A	C	1	0	0.77	0.23		orf19.886	C2_03380W_A	PAN1	Essential protein involved in endocytosis and polarized growth; ortholog of S. cerevisiae Pan1, which is a part of a complex that regulates actin cytoskeleton; Spider biofilm repressed
4043	D0	0 ug/mL FLC	SC5314	Synonymous	R	1561029	C	G	1	0	0.84	0.16		orf19.733	CR_07160C_A		Protein of unknown function
4043	D0	0 ug/mL FLC	SC5314	Synonymous	7	542549	G	A	1	0	0.92	0.08		orf19.6457	C7_02530C_A		Protein of unknown function
4043	D0	0 ug/mL FLC	SC5314	Synonymous	R	1898886	C	T	1	0	0.93	0.08		orf19.729.1	CR_07560W_A	RGD3	Putative Rho GTPase activating protein; fungal-specific (no human or murine homolog)
4043	D0	0 ug/mL FLC	SC5314	Missense	2	1710685	G	T	1	0	0.1	0.9	His530Asn	orf19.3631	C2_08470C_A	STN1	Protein involved in telomere maintenance; forms a complex with Ten1p
4043	D0	0 ug/mL FLC	SC5314	Missense	2	1709815	T	C	1	0	0.79	0.81	Asp122Gly	orf19.3633	C2_08460C_A		Ortholog(s) have role in purine nucleobase catabolic process
4043	D0	0 ug/mL FLC	SC5314	Missense	5	357465	C	G	0.99	0	0.57	0.43	Thr99Ile	orf19.6297	C5_01610W_A		Ortholog(s) have pseudouridine synthase activity, role in mRNA pseudouridine synthesis, tRNA pseudouridine synthesis and cytoplasm, nucleus localization
4043	D0	0 ug/mL FLC	SC5314	Missense	7	159700	T	C	1	0	0.92	0.08	Thr37Ala	orf19.7034	C7_00830C_A		Putative eIF4E-associated protein;, accelerates mRNA degradation by promoting decapping; Spider biofilm repressed
4044	E0	0 ug/mL FLC	SC5314	Synonymous	6	922714	C	T	1	0	0.85	0.15		orf19.73	C6_04180W_A		Putative metalloprotease; associates with ribosomes and is involved in ribosome biogenesis; Spider biofilm induced
4044	E0	0 ug/mL FLC	SC5314	Synonymous	4	60100	A	G	1	0	0.93	0.07		orf19.5666	C4_00390W_A		Protein of unknown function
4044	E0	0 ug/mL FLC	SC5314	Missense	3	91970	G	T	1	0	0.8	0.2	Gly1020Val	orf19.5404.1	C3_00580W_A	FLO9	Putative adhesin-like cell wall mannoprotein; repressed during the mating process; mutation confers hypersensitivity to toxic ergosterol analog; decreased transcription is observed upon fluphenazine treatment
4044	E0	0 ug/mL FLC	SC5314	Missense	5	466581	T	C	1	0	0.86	0.14	Asn59Ser	orf19.3160	C5_02080C_A	HSP12	Heat-shock protein; induced by osmotic/oxidative/cadmium stress, fluphenazine treatment, low iron, CDR1 and CDR2 overexpression, or ssn6 or ssk1 null mutation; overexpression increases resistance to farnesol and azoles
4044	E0	0 ug/mL FLC	SC5314	Missense	7	355295	T	C	1	0	0.85	0.13	Val251Ala	orf19.6559	C7_01650W_A		RNA polymerase III transcription initiation factor complex (TFIIIC) subunit; growth phase regulated protein; downregulaated in stationary phase yeast cultures; Hap43-repressed; flow model biofilm induced; Spider biofilm repressed
4044	E0	0 ug/mL FLC	SC5314	Missense	6	1008713	C	T	1	0	0.86	0.11	Arg1084Cys	orf19.2138	C6_04520W_A	ILS1	Putative isoleucyl-tRNA synthetase, the target of drugs including the cyclic beta-amino acid icofungipen/PLD-118/BAY-10–8888 and mupirocin; protein present in exponential and stationary growth phase yeast cultures
4044	E0	0 ug/mL FLC	SC5314	Missense	7	355297	G	A	1	0	0.89	0.11	Glu252Lys	orf19.6559	C7_01650W_A		RNA polymerase III transcription initiation factor complex (TFIIIC) subunit; growth phase regulated protein; downregulaated in stationary phase yeast cultures; Hap43-repressed; flow model biofilm induced; Spider biofilm repressed
4044	E0	0 ug/mL FLC	SC5314	Missense	1	1247580	T	A	1	0	0.9	0.1	Ile962Asn	orf19.2454	C1_05940W_A	PHO87	Putative phosphate permease; transcript repressed by Rim101 at pH 8; regulated by white-opaque switch; caspofungin repressed; virulence-group-correlated expression; flow model biofilm induced
4044	E0	0 ug/mL FLC	SC5314	Missense	1	711686	T	C	1	0	0.92	0.08	Phe719Leu	orf19.3038	C1_03380W_A	TPS2	Trehalose-6-phosphate (Tre6P) phosphatase; mutant heat sensitive, accumulates Tre6P, decreased mouse virulence; possible drug target; 2 conserved phospohydrolase motifs; no mammalian homolog; Hap43-repressed; flow model biofilm induced
4049	J0	0 ug/mL FLC	SC5314	Synonymous	R	1639813	G	A	1	0	0.86	0.14		orf19.729.1	CR_07560W_A	RGD3	Putative Rho GTPase activating protein; fungal-specific (no human or murine homolog)
4049	J0	0 ug/mL FLC	SC5314	Synonymous	5	526242	C	G	1	0	0.92	0.08		orf19.4245	C5_02370C_A		Protein with a predicted pleckstrin domain; Hap43-repressed gene
4049	J0	0 ug/mL FLC	SC5314	Missense	6	978106	C	T	1	0	0.88	0.13	Ala2218Val	orf19.1097	C6_04380W_A	ALS2	ALS family protein; role in adhesion, biofilm formation, germ tube induction; expressed at infection of human buccal epithelial cells; putative GPI-anchor; induced by ketoconazole, low iron and at cell wall regeneration; regulated by Sfu1p
4049	J0	0 ug/mL FLC	SC5314	Missense	3	1433325	A	G	1	0	0.89	0.11	Ile632Val	orf19.7403	C3_06290W_A		Ortholog of †S. cerevisiae †: YML020W, †C. glabrata CBS138 †: CAGL0G07062g, †C. dubliniensis CD36 †: Cd36_86190, †C. parapsilosis CDC317 †: CPAR2_404740 and †C. auris B8441 †: B9J08_000043
4049	J0	0 ug/mL FLC	SC5314	Missense	3	664683	C	A	1	0	0.92	0.08	Val66Leu	orf19.309	C3_03120C_A	DAL5	Allantoate permease; nitrogen catabolite repressed, induced in absence of preferred N sources; nitrogen source regulation requires Gat1; possibly essential gene (by UAU1 method); Hap43-repressed
4050	K0	0 ug/mL FLC	SC5314	Synonymous	2	614398	A	C	1	0	0.88	0.12		orf19.5797	C2_03040W_A	PLC2	Phosphatidylinositol (PtdIns)-specific phospholipase C (PI-PLC); predicted type 2 membrane protein; role in, and regulated by, filamentation, Nrg1 and Tup1; no mouse systemic virulence role; orf19.5797 and orf19.1586 are almost identical
4050	K0	0 ug/mL FLC	SC5314	Missense	1	1354674	A	T	1	0	0.88	0.12	Glu208Val	orf19.6275	C1_06360W_A		Protein of unknown function
4052	A1	1 ug/mL FLC	SC5314	Synonymous	1	3158906	T	C	1	0	0.54	0.46		orf19.7247	C1_14340C_A	RIM101	Transcription factor; alkaline pH response; required for alkaline-induced hyphal growth; role in virulence in mice; activated by C-terminal proteolytic cleavage; mediates both positive and negative regulation; Spider biofilm induced
4052	A1	1 ug/mL FLC	SC5314	Missense	1	3175359	T	C	1	0	0.94	0.06	Leu222Ser	orf19.7264	C1_14460W_A		Metalloprotease subunit of the 19S regulatory particle of the 26S proteasome lid; couples the deubiquitination and degradation of proteasome substrates; role in fission of mitochondria and peroxisome; Spider biofilm repressed
4053	B1	1 ug/mL FLC	SC5314	Missense	3	271154	A	C	1	0	0.8	0.14	Ser147Ala	orf19.1725	C3_01260C_A		Putative adhesin-like protein; highly expressed in white cells during pheromone response; required for adhesion, hyphal growth and biofilm formation
4055	D1	1 ug/mL FLC	SC5314	Synonymous	5	309133	T	C	1	0	0.61	0.39		orf19.1930	C5_01380W_A	CFL5	Ferric reductase; induced in low iron; ciclopirox olamine, flucytosine induced; amphotericin B, Sfu1 repressed; Tbf1, Hap43-induced
4055	D1	1 ug/mL FLC	SC5314	Missense	2	1773314	A	T	1	0	0.52	0.48	Asn348Ile	orf19.3604	C2_08720W_A		Ortholog(s) have Ino80 complex localization
4055	D1	1 ug/mL FLC	SC5314	Missense	R	155775	G	A	1	0	0.84	0.16	Gly191Glu	orf19.7466	CR_00640W_A	ACC1	Putative acetyl-coenzyme-A carboxylases; regulated by Efg1; amphotericin B repressed; caspofungin repressed; 5′-UTR intron; gene used for strain identification by multilocus sequence typing; Hap43-induced; flow model biofilm repressed †
4056	E1	1 ug/mL FLC	SC5314	Missense	2	2904127	G	A	1	0	0.46	0.54	Ser467Asn	orf19.4958	C1_13310W_A	ECM25	Non-essential protein involved in cell morphogenesis
4061	J1	1 ug/mL FLC	SC5314	Synonymous	3	118016	A	G	1	0	0.8	0.2		orf19.4173	C4_00690C_A		Protein of unknown function
4061	J1	1 ug/mL FLC	SC5314	Synonymous	R	1703810	A	G	1	0	0.94	0.06		orf19.3713	CR_07840C_A		Protein of unknown function; flow model biofilm induced; Spider biofilm induced; induced by Mnl1 under weak acid stress; transcript detected in high-resolution tiling arrays
4061	J1	1 ug/mL FLC	SC5314	Missense	2	1684776	A	T	1	0	0.8	0.2	His121Gln	orf19.1348	C2_08350C_A		Ortholog of †C. dubliniensis CD36 †: Cd36_22500, †Candida tropicalis NEW ASSEMBLY †: CTRG1_01825, †Candida tropicalis MYA-3404 †: CTRG_01825 and †Candida albicans WO-1 †: CAWG_05921
4062	K1	1 ug/mL FLC	SC5314	Synonymous	R	1986322	T	A	1	0	0.64	0.36		orf19.7332	CR_09370W_A	ELF1	Putative mRNA export protein; Walker A and B (ATP/GTP binding) motifs; required for wildtype morphology, growth; expressed in hyphal, pseudohyphal, and yeast form; Hap43-induced; Spider and flow model biofilm induced
4064	A8	8 ug/mL FLC	SC5314	Synonymous	2	2905332	T	C	1	0	0.44	0.52		orf19.4949	C1_13210C_A		Protein of unknown function
4064	A8	8 ug/mL FLC	SC5314	Synonymous	R	1641968	G	A	1	0	0.88	0.13		orf19.729.1	CR_07560W_A	RGD3	Putative Rho GTPase activating protein; fungal-specific (no human or murine homolog)
4067	D8	8 ug/mL FLC	SC5314	Missense	1	1750316	A	T	1	0	0.83	0.17	Tyr6Phe	orf19.5073	C1_08010W_A	DPM1	Dolichol-phosphate mannose synthase catalytic subunit; filament induced; Tup1-regulated; flow model and rat catheter biofilm repressed
4068	E8	8 ug/mL FLC	SC5314	Missense	1	3036771	C	T	1	0	0.5	0.5	Ala504Val	orf19.5019	C1_13810W_A		Ortholog of †C. dubliniensis CD36 †: Cd36_12800, †C. parapsilosis CDC317 †: CPAR2_203460, †C. auris B8441 †: B9J08_004645 and †Candida tenuis NRRL Y-1498 †: CANTEDRAFT_94106 †
4073	J8	8 ug/mL FLC	SC5314	Missense	3	1638971	A	T	0.99	0	0.91	0.09	Thr323Ser	orf19.6784	C3_07160W_A	PGA32	Putative GPI-anchored adhesin-like protein; induced in high iron; Spider biofilm induced
4076	A64	64 ug/mL FLC	SC5314	Missense	3	766113	C	A	1	0	0.9	0.1	Ala65Asp	orf19.6958	C3_03630W_A	ECM18	Ortholog of †S. cerevisiae †: ECM18, †C. glabrata CBS138 †: CAGL0B01969g, †C. parapsilosis CDC317 †: CPAR2_103190, †C. auris B8441 †: B9J08_000758 and †Debaryomyces hansenii CBS767 †: DEHA2G08448g
4076	A64	64 ug/mL FLC	SC5314	Missense	R	768035	A	G	1	0	0.91	0.09	Ser175Gly	orf19.4390	CR_03470W_A		Protein of unknown function; repressed by alpha pheromone in SpiderM medium; transcript induced by Mnl1 under weak acid stress
4077	B64	64 ug/mL FLC	SC5314	Synonymous	2	525945	A	C	1	0	0.59	0.41		orf19.1586	C2_02600C_A	FGR22	Putative phosphatidylinositol-specific phospholipase C (PI-PLC); predicted type 2 membrane protein; no S. cerevisiae ortholog; role in, and regulated by, filamentation, Hap43p; almost identical to orf19.5797
4077	B64	64 ug/mL FLC	SC5314	Synonymous	6	632320	T	C	1	0	0.69	0.31		orf19.5584	C6_03020W_A	PEP3	Peptidase; activity useful for strain identification by multilocus enzyme electrophoresis (MLEE); clade-associated gene expression
4077	B64	64 ug/mL FLC	SC5314	Synonymous	6	5640	A	G	1	0	0.903	0.07		orf19.6337	C6_00030W_A	TLO13	Member of a family of telomere-proximal genes of unknown function; may be spliced in vivo; overlaps orf19.6337.1, which is a region annotated as blocked reading frame
4079	D64	64 ug/mL FLC	SC5314	Nonsense	1	261682	G	A	0.99	0	0.82	0.18	Gln56*	orf19.3322	C1_01330C_A	DUT1	dUTP pyrophosphatase; cell-cycle regulated if expressed in S. cerevisiae; upstream MluI and SCB elements; 17-beta-estradiol, ethynyl estradiol, macrophage induced; decreased in stationary phase yeast; rat catheter, Spider biofilm repressed
4085	J64	64 ug/mL FLC	SC5314	Synonymous	2	1245148	T	C	1	0	0.87	0.13		orf19.4110	C2_06090W_A		Protein of unknown function
4086	K64	64 ug/mL FLC	SC5314	Synonymous	R	101986	A	C	1	0	0.78	0.22		orf19.7494	CR_00390W_A	MMS22	Protein of unknown function; cell-cycle regulated periodic mRNA expression
4091	P0	0 ug/mL FLC	P75063	Missense	4	617622	T	C	1	0	0.29	0.71	Lys1015Arg	orf19.2695	C4_02970C_A	UBR1	Protein similar to S. cerevisiae Ubr1p ubiquitin-protein ligase; transposon mutation affects filamentous growth; Spider biofilm induced
4091	P0	0 ug/mL FLC	P75063	Missense	3	878550	C	T	1	0	0.66	0.34	Arg312Stp	orf19.5865	C3_04230W_A		Ortholog(s) have RNA-dependent ATPase activity, role in generation of catalytic spliceosome for first transesterification step and U2-type catalytic step 1 spliceosome localization
4091	P0	0 ug/mL FLC	P75063	Missense	4	1176506	T	A	0.93	0	0.72	0.28	Asn355Ile	orf19.1797	C4_05400C_A		D-arabinose 5-phosphate isomerase; has GutQ domain which is associated with phosphosugar binding; other biofilm induced; rat catheter and Spider biofilm induced; F-12/CO2 early biofilm induced
4092	Q0	0 ug/mL FLC	P75063	Missense	5	152008	T	G	1	0	0.31	0.69	Asn409Thr	orf19.921	C5_00670C_A	HMS1	hLh domain Myc-type transcript factor; required for morphogenesis induced by elevated temperature or Hsp90 compromise; acts downstream of Pcl1; Spider biofilm induced
4092	Q0	0 ug/mL FLC	P75063	Missense	R	179271	G	C	1	0	0.7	0.3	Thr736Ser	orf19.3279	CR_00760C_A	HYR4	Putative GPI-anchored adhesin-like protein; Rim101-repressed; constitutive expression independent of MTL or white-opaque status
4092	Q0	0 ug/mL FLC	P75063	Missense	7	178097	A	T	1	0	0.85	0.15	Leu408Met	orf19.7027	C7_00880C_A		Protein of unknown function; Spider biofilm induced
4093	R0	0 ug/mL FLC	P75063	Synonymous	3	426679	T	C	1	0	0.88	0.12		orf19.1655	C3_01930W_A	PXP2	Putative acyl-CoA oxidase; enzyme of fatty acid beta-oxidation; induced during macrophage infection; opaque specific transcript; putative peroxisome targeting signal; Spider biofilm induced
4093	R0	0 ug/mL FLC	P75063	Missense	4	608041	A	C	1	0	0.85	0.15	Lys482Asn	orf19.2699	C4_02940W_A	ABP1	Ortholog of S. cerevisiae Abp1; actin-binding protein of the cortical actin cytoskeleton; caspofungin induced; protein only detected in stationary phase yeast-form cultures; Spider biofilm repressed
4094	S0	0 ug/mL FLC	P75063	Synonymous	6	491030	A	G	1	0	0.84	0.16		orf19.3476	C6_02340W_A	HRR25	Predicted protein serine–threonine kinase; Spider biofilm induced
4094	S0	0 ug/mL FLC	P75063	Synonymous	7	542558	A	G	1	0	0.87	0.13		orf19.6457	C7_02530C_A		Ortholog of S. cerevisiae: YBL086C, C. glabrata CBS138 : CAGL0C01815g, C. dubliniensis CD36 : Cd36_72270, C. parapsilosis CDC317 : CPAR2_702850 and Candida tenuis NRRL Y-1498 : CANTEDRAFT_107537
4094	S0	0 ug/mL FLC	P75063	Missense	1	78768	C	G	1	0	0.81	0.19	Trp301Cys	orf19.6058	C1_00500C_A	GLO1	Putative monomeric glyoxalase I; oxidative stress-induced via Cap1; flow model and rat catheter biofilm repressed
4094	S0	0 ug/mL FLC	P75063	Missense	4	341502	G	A	1	0	0.83	0.17	Ala208Val	orf19.4622	C4_01720C_A		Ortholog(s) have role in telomere maintenance, transcription-coupled nucleotide-excision repair, ubiquitin-dependent protein catabolic process and nucleus localization
4094	S0	0 ug/mL FLC	P75063	Missense	4	1335653	T	C	1	0	0.85	0.15	Asn770Asp	orf19.4412	C4_06020C_A		Ortholog(s) have DNA-directed DNA polymerase activity, deoxycytidyl transferase activity and role in error-free translesion synthesis, error-prone translesion synthesis
4094	S0	0 ug/mL FLC	P75063	Missense	3	1182045	T	A	1	0	0.88	0.13	Ser303Cys	orf19.6970	C3_05330C_A		Ortholog of C. dubliniensis CD36 : Cd36_85310, C. parapsilosis CDC317 : CPAR2_807370, Candida tenuis NRRL Y-1498 : CANTEDRAFT_115544 and Debaryomyces hansenii CBS767 : DEHA2D11770g
4094	S0	0 ug/mL FLC	P75063	Missense	R	500124	T	G	0.99	0.01	0.87	0.13	Asn861Thr	orf19.3746	CR_02240C_A	OPT2	Oligopeptide transporter; induced upon phagocytosis by macrophage; macrophage/pseudohyphal-repressed after 16 h; fluconazole-induced; virulence-group-correlated expression; Hap43-repressed
4094	S0	0 ug/mL FLC	P75063	Missense	3	1615748	A	G	1	0	0.88	0.12	Asn74Ser	orf19.6795	C3_07050W_A		Ortholog(s) have enzyme activator activity
4094	S0	0 ug/mL FLC	P75063	Missense	2	1241761	T	G	1	0	0.89	0.11	Lys237Asn	orf19.4112	C2_06080C_A		Ortholog(s) have 8-oxo-dGDP phosphatase activity, ATP binding, magnesium ion binding, thiamin binding, thiamin diphosphokinase activity, role in thiamin biosynthetic process and cytosol localization
4094	S0	0 ug/mL FLC	P75063	Missense	2	2177573	A	T	1	0	0.9	0.1	Phe598Tyr	orf19.5328	C2_10550C_A	GCN1	Ortholog(s) have role in regulation of translational elongation and cytosolic ribosome, extracellular region, mitochondrion localization
4103	P1	1 ug/mL FLC	P75063	Missense	R	1120281	A	C	1	0	0.79	0.21	Val54Gly	orf19.637	CR_05180C_A	SDH2	Succinate dehydrogenase, Fe-S subunit; localizes to surface of yeast cells, but not hyphae; induced in high iron and during log phase aerobic growth; repressed by nitric oxide, Hap43
4104	Q1	1 ug/mL FLC	P75063	Synonymous	1	1386929	A	G	1	0	0.8	0.2		orf19.6261	C1_06520C_A	BPH1	Ortholog of S. cerevisiae Bph1; a putative ortholog of human Chediak-Higashi syndrome protein and murine beige gene implicated in disease syndromes involving defective lysosomal trafficking; mutant is viable
4104	Q1	1 ug/mL FLC	P75063	Synonymous	6	718826	C	T	1	0	0.84	0.16		orf19.5701	C6_03420W_A		Ortholog(s) have role in DNA replication initiation, establishment of mitotic sister chromatid cohesion and condensed nuclear chromosome kinetochore localization
4104	Q1	1 ug/mL FLC	P75063	Synonymous	5	702284	T	A	0.99	0	0.85	0.15		orf19.4346	C5_03140C_A		Ortholog(s) have protein anchor activity, role in COPII vesicle coating, protein localization to endoplasmic reticulum exit site and ER to Golgi transport vesicle membrane, endoplasmic reticulum exit site localization
4104	Q1	1 ug/mL FLC	P75063	Synonymous	3	1301132	A	G	1	0	0.89	0.11		orf19.7362	C3_05810C_A	SKN1	Protein with a role in beta-1,6-glucan synthesis; probable N-glycosylated type II membrane protein; transcript and mRNA length change induced by yeast-hypha transition; induced by Rim101, caspofungin; rat catheter and Spider biofilm induced
4104	Q1	1 ug/mL FLC	P75063	Nonsense	3	1434670	G	T	1	0	0.9	0.1	Glu127Stp	orf19.7402	C3_06300W_A	DOT1	Putative modulator of white-opaque switching
4104	Q1	1 ug/mL FLC	P75063	Missense	R	1022283	T	A	1	0	0.53	0.47	Lys1106Asn	orf19.1734	CR_04720C_A		Putative ATPase and nucleosome spacing factor; heterozygous null mutant displays sensitivity to virgineone
4104	Q1	1 ug/mL FLC	P75063	Missense	1	919542	C	A	1	0	0.78	0.22	Glu26Asp	orf19.6838	C1_04460C_A		Putative protein of unknown function, transcript upregulated in clinical isolates from HIV + patients with oral candidiasis; Spider biofilm induced
4104	Q1	1 ug/mL FLC	P75063	Missense	6	770861	A	C	1	0	0.8	0.2	Asp184Glu	orf19.5730	C6_03620C_A		Putative phenylacrylic acid decarboxylase; clade-associated gene expression
4104	Q1	1 ug/mL FLC	P75063	Missense	2	2099776	T	C	1	0	0.85	0.15	Ser271Gly	orf19.1759	C2_10220C_A	PHO23	Ortholog(s) have methylated histone residue binding activity
4104	Q1	1 ug/mL FLC	P75063	Missense	R	1918281	C	T	1	0	0.87	0.13	Glu232Lys	orf19.7293	CR_08960C_A	MPS1	Monopolar spindle protein, a putative kinase; essential for growth; periodic mRNA expression, peak at cell-cycle S/G2 phase
4104	Q1	1 ug/mL FLC	P75063	Missense	7	416284	T	A	0.99	0	0.89	0.11	Asp110Val	orf19.6527	C7_01940C_A		Pheromone-regulated protein (Prm10) of S. cerevisiae; colony morphology-related gene regulation by Ssn6; induced by Mnl1 under weak acid stress; possibly essential gene, disruptants not obtained by UAU1 method; Spider biofilm induced
4105	R1	1 ug/mL FLC	P75063	Synonymous	3	548519	G	A	1	0	0.88	0.13		orf19.242	C3_02510C_A	SAP8	Secreted aspartyl protease; regulated by growth phase, temperature, white-opaque switch; highly expressed in opaque cells and upon deep epidermal invasion; greater expression in vaginal than oral infection; prominent role in biofilms
4105	R1	1 ug/mL FLC	P75063	Synonymous	4	72816	G	T	1	0	0.89	0.11		orf19.5674	C4_00450C_A	PGA10	GPI-anchored membrane protein; utilization of hemin and hemoglobin for Fe in host; Rim101 at ph8/hypoxia/ketoconazole/ciclopirox/hypha-induced; required for RPMI biofilm formation, Bcr1-induced in a/a biofilm; rat catheter biofilm repressed
4106	S1	1 ug/mL FLC	P75063	Synonymous	R	1707424	A	G	1	0	0.7	0.3		orf19.3714	CR_07850W_A		Ortholog of C. dubliniensis CD36 : Cd36_33530, C. parapsilosis CDC317 : CPAR2_201980, Candida tenuis NRRL Y-1498 : CANTEDRAFT_134293 and Debaryomyces hansenii CBS767 : DEHA2A10164g
4106	S1	1 ug/mL FLC	P75063	Synonymous	R	726132	A	G	1	0	0.85	0.15		orf19.2400	CR_03230W_A		Ortholog(s) have role in mRNA splicing, via spliceosome, maturation of SSU-rRNA, positive regulation of ATPase activity, positive regulation of helicase activity
4106	S1	1 ug/mL FLC	P75063	Missense	5	1060308	A	G	1	0	0.86	0.14	Phe42Leu	orf19.3972	C5_04860C_A		Ortholog(s) have role in ER to Golgi vesicle-mediated transport, Golgi to endosome transport and ER to Golgi transport vesicle, Golgi membrane, endoplasmic reticulum localization
4106	S1	1 ug/mL FLC	P75063	Missense	7	484084	G	A	1	0	0.89	0.11	Ser318Leu	orf19.6492	C7_02220C_A		Predicted protein serine–threonine kinase and/or protein tyrosine kinase; Spider biofilm induced
4106	S1	1 ug/mL FLC	P75063	Missense	R	562113	C	G	1	0	0.89	0.11	Arg61Gly	orf19.171	CR_02530W_A	DBP2	Putative DEAD-box family ATP-dependent RNA helicase; flucytosine induced; repressed in core stress response
4106	S1	1 ug/mL FLC	P75063	Missense	3	426357	T	A	1	0	0.9	0.1	Phe343Tyr	orf19.1655	C3_01930W_A	PXP2	Putative acyl-CoA oxidase; enzyme of fatty acid beta-oxidation; induced during macrophage infection; opaque specific transcript; putative peroxisome targeting signal; Spider biofilm induced
4107	T1	1 ug/mL FLC	P75063	Synonymous	1	2118448	G	A	0.99	0	0.83	0.15		orf19.4824	C1_09610W_A		Planktonic growth-induced gene
4107	T1	1 ug/mL FLC	P75063	Missense	1	3077300	T	G	1	0	0	1	Phe188Val	orf19.5034	C1_13960W_A	YBP1	Protein involved in response to oxidative stress, binds and stabilizes Cap1p transcription factor in response to H_2_O_2_; essential for macrophage killing
4107	T1	1 ug/mL FLC	P75063	Missense	1	1845690	T	A	1	0	0.48	0.52	Gln68Ser	orf19.384	C1_08390C_A		Ortholog(s) have Golgi apparatus, cytosol, nucleus localization
4107	T1	1 ug/mL FLC	P75063	Missense	2	328914	A	T	1	0	0.63	0.37	Thr406Ser	orf19.1490	C2_01780W_A	MSB2	Mucin family adhesin-like protein; cell wall damage sensor; required for Cek1 phosphorylation by cell wall stress; Rim101-repressed; activation releases extracellular domain into medium; Spider biofilm induced
4111	X1	1 ug/mL FLC	P75063	Synonymous	3	1307688	G	A	1	0	0.83	0.17		orf19.7363	C3_05830W_A	KRE6	Essential beta-1,6-glucan synthase subunit; change in mRNA length, not abundance, at yeast-hypha transition; alkaline-induced by Rim101, on cell wall regeneration; Spider biofilm induced; Bcr1-repressed in RPMI a/a biofilms
4111	X1	1 ug/mL FLC	P75063	Synonymous	1	2802671	C	T	1	0	0.85	0.15		orf19.4917	C1_12870C_A		Ortholog of Candida albicans WO-1 : CAWG_00155
4111	X1	1 ug/mL FLC	P75063	Synonymous	3	431160	G	A	1	0	0.86	0.14		orf19.1652	C3_01960C_A	POX1-3	Predicted acyl-CoA oxidase; farnesol regulated; stationary phase enriched protein; Spider biofilm induced
4111	X1	1 ug/mL FLC	P75063	Synonymous	3	1307307	C	T	1	0	0.89	0.11		orf19.7363	C3_05830W_A	KRE6	Essential beta-1,6-glucan synthase subunit; change in mRNA length, not abundance, at yeast-hypha transition; alkaline-induced by Rim101, on cell wall regeneration; Spider biofilm induced; Bcr1-repressed in RPMI a/a biofilms
4115	P8	8 ug/mL FLC	P75063	Synonymous	R	2265393	T	A	1	0	0.83	0.17		orf19.7655	CR_10680W_A	RPO21	RNA polymerase II; ortholog of S. cerevisiae Rpo21, transposon mutation affects filamentous growth; flow model biofilm repressed
4115	P8	8 ug/mL FLC	P75063	Missense	3	1550473	A	G	1	0	0.56	0.44	Lys459Arg	orf19.6824	C3_06790W_A	TRY6	Helix-loop-helix transcription factor; regulator of yeast form adherence; required for yeast cell adherence to silicone substrate; Spider and F-12/CO2 biofilm induced; repressed by alpha pheromone in SpiderM medium
4115	P8	8 ug/mL FLC	P75063	Missense	3	257365	C	A	1	0	0.62	0.38	Thr2436Asn	orf19.3159	C3_01200W_A	UTP20	Putative snoRNA-binding protein; S. cerevisiae Utp20 ortholog; likely essential for growth; repressed in core stress response; mutation confers resistance to 5-fluorocytosine (5-FC) and parnafungin
4115	P8	8 ug/mL FLC	P75063	Missense	3	1182192	C	G	1	0	0.68	0.32	Ala254Pro	orf19.6970	C3_05330C_A		Ortholog of C. dubliniensis CD36 : Cd36_85310, C. parapsilosis CDC317 : CPAR2_807370, Candida tenuis NRRL Y-1498 : CANTEDRAFT_115544 and Debaryomyces hansenii CBS767 : DEHA2D11770g
4115	P8	8 ug/mL FLC	P75063	Missense	R	111103	T	G	1	0	0.78	0.22	Gln510His	orf19.7489	CR_00440C_A	LRG1	Ortholog(s) have Rho GTPase activator activity
4116	Q8	8 ug/mL FLC	P75063	Synonymous	6	887441	G	A	1	0	0.38	0.62		orf19.1214	C6_04080W_A		Ortholog(s) have metalloaminopeptidase activity, role in protein initiator methionine removal involved in protein maturation and cytosol, nucleus localization
4116	Q8	8 ug/mL FLC	P75063	Missense	5	187064	C	A	1	0	0.84	0.16	Gly393Val	orf19.581	C5_00790C_A		Putative RNA-binding protein; transcript is upregulated in an RHE model of oral candidiasis
4117	R8	8 ug/mL FLC	P75063	Synonymous	2	2162314	G	A	1	0	0.83	0.17		orf19.5318	C2_10440C_A	RAD1	Putative single-stranded DNA endonuclease; transcript regulated by Nrg1; macrophage-induced gene
4117	R8	8 ug/mL FLC	P75063	Synonymous	3	324794	A	T	1	0	0.87	0.13		orf19.1693	C3_01530C_A	CAS4	RAM cell wall integrity signaling network protein; cell separation, azole sensitivity; needed for hyphal growth; insertion mutation near 3′ end of gene increases caspofungin sensitivity; pheromone/hyphal induced; flow biofilm repressed
4117	R8	8 ug/mL FLC	P75063	Nonsense	2	978178	A	T	1	0	0.78	0.22	Tyr385Stp	orf19.147	C2_04660C_A	YAK1	Predicted serine–threonine protein kinase; involved in hyphal growth regulation and biofilm formation; flow model biofilm induced; induced in core caspofungin response
4117	R8	8 ug/mL FLC	P75063	Missense	6	585033	C	T	1	0	0.83	0.17	Ala451Val	orf19.5557	C6_02830W_A	MNN4-4	Mannosyltransferase; transcript upregulated in Ssk1 response regulator mutant or in nik1 null mutant, but not in chk1 or sln1 null mutants; pheromone induced; Spider biofilm induced
4117	R8	8 ug/mL FLC	P75063	Missense	R	2002065	A	T	1	0	0.86	0.14	Ile556Leu	orf19.7337	CR_09410W_A		Protein with a nischarin related domain and leucine rich repeats; Spider biofilm induced
4119	T8	8 ug/mL FLC	P75063	Missense	2	754110	G	A	1	0	0.83	0.17	Ser299Phe	orf19.864	C2_03550C_A		Ortholog(s) have role in nuclear-transcribed mRNA catabolic process, 3'-5” exonucleolytic nonsense-mediated decay and cytosol, polysome localization
4123	X8	8 ug/mL FLC	P75063	Synonymous	1	2808396	A	G	1	0	0.89	0.11		orf19.4921	C1_12900W_A		Ortholog of C. dubliniensis CD36 : Cd36_12030 and Candida albicans WO-1 : CAWG_00152
4123	X8	8 ug/mL FLC	P75063	Synonymous	R	1156300	T	A	1	0	0.89	0.11		orf19.5286	CR_05380C_A	YCP4	Putative flavodoxin; flow model, rat catheter and Spider biofilm repressed
4123	X8	8 ug/mL FLC	P75063	Missense	6	342123	A	C	1	0	0.65	0.35	Leu1981Arg	orf19.3422	C6_01650C_A	FMP27	Putative mitochondrial protein; mRNA binds She3
4129	R64	64 ug/mL FLC	P75063	Missense	7	379336	T	G	1	0	0.71	0.29	Val99Gly	orf19.6547	C7_01770W_A		Ortholog of Candida albicans WO-1 : CAWG_05528
4130	S64	64 ug/mL FLC	P75063	Synonymous	2	1772283	T	A	1	0	0.81	0.19		orf19.3604	C2_08720W_A		Ortholog(s) have nucleus localization
4130	S64	64 ug/mL FLC	P75063	Missense	4	317376	A	T	1	0	0.5	0.5	His274Gln	orf19.4631	C4_01530C_A	ERG251	C-4 sterol methyl oxidase; role in ergosterol biosynthesis; Hap43-induced; ketoconazole-induced; amphotericin B, caspofungin repressed; possibly essential gene, disruptants not obtained by UAU1 method; Spider biofilm repressed
4130	S64	64 ug/mL FLC	P75063	Missense	1	478761	G	A	0.99	0.01	0.71	0.29	Thr948Ile	orf19.3685	C1_02280C_A	PSY2	Putative protein phosphatase PP4 complex subunit; macrophage-induced gene
4130	S64	64 ug/mL FLC	P75063	Missense	2	353976	C	A	1	0	0.84	0.16	His1082Gln	orf19.1499	C2_01890W_A	CTF1	Putative zinc-finger transcription factor, similar to A. nidulans FarA and FarB; activates genes required for fatty acid degradation; induced by oleate; null mutant displays carbon source utilization defects and slightly reduced virulence
4131	T64	64 ug/mL FLC	P75063	Missense	1	3158830	A	G	1	0	0.81	0.19	Phe610Leu	orf19.7247	C1_14340C_A	RIM101	Transcription factor; alkaline pH response; required for alkaline-induced hyphal growth; role in virulence in mice; activated by C-terminal proteolytic cleavage; mediates both positive and negative regulation; Spider biofilm induced
4135	X64	64 ug/mL FLC	P75063	Synonymous	1	1903411	G	A	1	0	0.66	0.29		orf19.4731	C1_08730W_A		Ortholog(s) have role in CVT pathway, intra-Golgi vesicle-mediated transport and Golgi transport complex localization
4194	K0	0 ug/mL FLC	FH1	Synonymous	6	924426	G	A	1	0	0.74	0.26		orf19.1075	C6_04190C_A		Protein of unknown function; Spider biofilm induced
4195	L0	0 ug/mL FLC	FH1	Synonymous	4	1624	T	C	1	0	0.94	0.06		orf19.362	C4_00010W_A	TLO9	Member of a family of telomere-proximal genes of unknown function; Hap43p-repressed gene
4195	L0	0 ug/mL FLC	FH1	Synonymous	6	799393	T	C	1	0	0.93	0.06		orf19.5742	C6_03710W_A	ALS9	ALS family cell-surface glycoprotein; expressed during infection of human epithelial cells; confers laminin adhesion to S. cerevisiae; highly variable; putative GPI-anchor; Hap43-repressed
4199	D1	1 ug/mL FLC	FH1	Synonymous	R	562329	G	A	1	0	0.58	0.42		orf19.5543	C6_02720C_A		Ortholog(s) have cell division site, cytosol localization
4199	D1	1 ug/mL FLC	FH1	Missense	4	1205158	T	C	1	0	0.81	0.19	Trp129Arg	orf19.1234	C4_05530W_A	FGR6-10	Protein lacking an ortholog in S. cerevisiae; member of a family encoded by FGR6-related genes in the RB2 repeat sequence; transposon mutation affects filamentous growth
4200	E1	1 ug/mL FLC	FH1	Synonymous	2	932372	C	T	1	0	0.74	0.26		orf19.4506	C2_04460W_A	LYS22	Putative homocitrate synthase; repressed by nitric oxide and by hypoxia; protein level decreases in stationary phase cultures; induced by ketoconazole, Spider biofilm induced; flow model biofilm repressed
4201	F1	1 ug/mL FLC	FH1	Synonymous	2	1358178	A	G	1	0	0.94	0.05		orf19.1223	C2_06670C_A	DBF2	Essential serine–threonine protein kinase involved in mitotic spindle formation and cytokinesis; required for septum formation, exit from mitosis, and normal hyphal morphogenesis; virulence-group-correlated expression
4204	I1	1 ug/mL FLC	FH1	Synonymous	4	1624	T	C	1	0	0.96	0.04		orf19.362	C4_00010W_A	TLO9	Member of a family of telomere-proximal genes of unknown function; Hap43p-repressed gene
4204	I1	1 ug/mL FLC	FH1	Missense	7	216768	T	C	1	0	0.92	0.06	Asn604Ser	orf19.7011	C7_01030C_A		Ortholog(s) have 90S preribosome, cytoplasm, mitotic spindle pole body, nucleolus localization
4206	K1	1 ug/mL FLC	FH1	Synonymous	2	164778	C	T	1	0	0.9	0.1		orf19.2023	C2_01000W_A		Putative MFS glucose transporter; glucose, fluconazole, Snf3 induced, expressed at high glucose; 20 member C. albicans glucose transporter family; 12 TM regions predicted; flow model biofilm induced; Spider biofilm repressed
4206	K1	1 ug/mL FLC	FH1	Missense	2	164780	CT	TG	1	0	0.90, 0.86	0.10, 0.14	Ala105Val	orf19.2023	C2_01000W_A	HGT7	Putative MFS glucose transporter; glucose, fluconazole, Snf3 induced, expressed at high glucose; 20 member C. albicans glucose transporter family; 12 TM regions predicted; flow model biofilm induced; Spider biofilm repressed
4208	A8	8 ug/mL FLC	FH1	Synonymous	4	1162082	A	T	1	0	0.95	0.05		orf19.1792	C4_05340W_A		Ortholog(s) have ubiquitin-protein ligase activity
4208	A8	8 ug/mL FLC	FH1	Missense	4	1162092	A	T	1	0	0.94	0.06	Thr18Ser	orf19.1792	C4_05340W_A		Ortholog(s) have ubiquitin-protein ligase activity
4209	B8	8 ug/mL FLC	FH1	Missense	2	968404	T	C	1	0	0.95	0.05	Ile968Thr	orf19.4488	C2_04620W_A		Predicted ortholog of S. cerevisiae Swi3, subunit of the SWI/SNF chromatin remodeling complex; possibly an essential gene, disruptants not obtained by UAU1 method
4210	C8	8 ug/mL FLC	FH1	Synonymous	2	170933	A	T	1	0	0.94	0.06		orf19.2020	C2_01020W_A	HGT6	Putative high-affinity MFS glucose transporter; 20 family members; induced in core stress response; fluconazole, oralpharyngeal candidasis induced; flow model biofilm induced; Spider biofilm induced
4211	D8	8 ug/mL FLC	FH1	Synonymous	1	1759869	T	C	1	0	0.84	0.16		orf19.5079	C1_08070W_A	CDR4	Putative ABC transporter superfamily; fluconazole, Sfu1, Hog1, core stress response induced; caspofungin repressed; fluconazole resistance not affected by mutation or correlated with expression; rat catheter and flow model biofilm induced
4211	D8	8 ug/mL FLC	FH1	Missense	R	1948195	G	A	1	0	0.71	0.29	Ala608Val	orf19.7310	CR_09140C_A		Protein with a role in directing meiotic recombination events to homologous chromatids; induced by ciclopirox olamine; positively regulated by Sfu1; Hog1, fluconazole-repressed; Hap43-induced; Spider biofilm induced
4211	D8	8 ug/mL FLC	FH1	Missense	7	586612	A	G	1	0	0.87	0.13	Tyr384Cys	orf19.5191	C7_02750W_A	FGR6-1	Protein lacking an ortholog in S. cerevisiae; member of a family encoded by FGR6-related genes in the RB2 repeat sequence; transposon mutation affects filamentous growth
4214	G8	8 ug/mL FLC	FH1	Missense	3	223252	C	A	1	0	0.89	0.11	Pro115His	orf19.2512	C3_01100W_A		Ortholog of C. dubliniensis CD36 : Cd36_81000, C. parapsilosis CDC317 : CPAR2_103050, Candida tenuis NRRL Y-1498 : CANTEDRAFT_116326 and Debaryomyces hansenii CBS767 : DEHA2G01958g
4215	H8	8 ug/mL FLC	FH1	Synonymous	6	972803	T	C	1	0	0.6	0.4		orf19.1097	C6_04380W_A	ALS2	ALS family protein; role in adhesion, biofilm formation, germ tube induction; expressed at infection of human buccal epithelial cells; putative GPI-anchor; induced by ketoconazole, low iron and at cell wall regeneration; regulated by Sfu1p
4216	I8	8 ug/mL FLC	FH1	Synonymous	4	1492501	A	G	1	0	0.9	0.1		orf19.3141	C4_06680C_A		Ortholog(s) have role in ER to Golgi vesicle-mediated transport and cytoplasmic mRNA processing body, endoplasmic reticulum membrane, extrinsic to membrane localization
4217	J8	8 ug/mL FLC	FH1	Synonymous	1	2894825	T	C	1	0	0.88	0.1		orf19.4953	C1_13270W_A		Putative ATPase; predicted role in ER-associated protein catabolism; induced during chlamydospore formation in both C. albicans and C. dubliniensis; rat catheter biofilm repressed
4217	J8	8 ug/mL FLC	FH1	Missense	3	1449395	A	G	1	0	0.81	0.14	Ile929Val	orf19.7400	C3_06320W_A	ALS7	ALS family protein; hypermutable contingency gene; growth-regulated, downregulated in biofilm; two variable repeat regions; expression in S. cerevisiae does not confer adhesiveness; ALS family includes adhesins, cell-surface glycoproteins
4217	J8	8 ug/mL FLC	FH1	Missense	1	2750212	C	A	1	0	0.94	0.06	Pro140Gln	orf19.3755	C1_12610W_A		Ortholog(s) have structural constituent of ribosome activity and mitochondrial large ribosomal subunit localization
4218	K8	8 ug/mL FLC	FH1	Synonymous	6	1031244	G	A	1	0	0.85	0.14		orf19.2163	C6_04650W_A		Ortholog(s) have cytosol localization
4218	K8	8 ug/mL FLC	FH1	Synonymous	2	1495724	G	A	1	0	0.9	0.08		orf19.2290	C2_07330W_A	TOR1	Protein similar to TOR family phosphatidylinositol kinases; mutation confers resistance to rapamycin; involved in regulation of ribosome biogenesis, starvation response, and adhesion
4218	K8	8 ug/mL FLC	FH1	Missense	4	635223	G	T	1	0	0.92	0.08	Asp166Glu	orf19.2686	C4_03050C_A		Ortholog(s) have carboxypeptidase activity, role in nitrogen compound metabolic process, proteolysis involved in cellular protein catabolic process and fungal-type vacuole lumen localization
4221	B64	64 ug/mL FLC	FH1	Synonymous	2	888419	A	G	1	0	0.9	0.08		orf19.797	C2_04230W_A	BAT21	Putative branched chain amino acid aminotransferase; regulated by Gcn4, Gcn2; induced in response to amino acid starvation (3-aminotriazole treatment); early-stage flow model biofilm formation
4222	C64	64 ug/mL FLC	FH1	Synonymous	1	2801565	T	C	1	0	0.91	0.09		orf19.4916	C1_12860C_A		Protein of unknown function; induced by alpha pheromone in SpiderM medium
4222	C64	64 ug/mL FLC	FH1	Synonymous	1	2801568	C	T	1	0	0.91	0.09		orf19.4916	C1_12860C_A		Protein of unknown function; induced by alpha pheromone in SpiderM medium
4224	E64	64 ug/mL FLC	FH1	Missense	5	401422	G	A	1	0	0.91	0.09	Ala305Thr	orf19.3197	C5_01780W_A	PAP1	Poly(A) polymerase, likely involved in mRNA polyadenylation; PAP is inhibited by parnafungin antifungals; non-sex gene located within the MTLa mating-type-like locus
4225	F64	64 ug/mL FLC	FH1	Missense	3	13936	C	A	1	0	0.93	0.06	His61Asn	orf19.5467	C3_00060W_A	TLO7	Member of a family of telomere-proximal genes of unknown function; may be spliced in vivo; rat catheter biofilm repressed
4226	G64	64 ug/mL FLC	FH1	Missense	R	1066847	A	G	1	0	0.38	0.62	Asn216Ser	orf19.1805	CR_04930W_A	PEX14	Ortholog(s) have peroxisome matrix targeting signal-1 binding, peroxisome matrix targeting signal-2 binding, protein binding, bridging activity
4227	H64	64 ug/mL FLC	FH1	Synonymous	1	1920491	A	C	1	0	0.89	0.12		orf19.4739	C1_08810C_A	MSS116	Putative DEAD-box protein; required for efficient splicing of mitochondrial Group I and II introns; Hap43-induced; rat catheter biofilm induced
4228	I64	64 ug/mL FLC	FH1	Synonymous	R	2151160	G	C	1	0	0.86	0.14		orf19.7581	CR_10060W_A		Protein with a predicted role in assembly of U2 snRNP into the spliceosome; Spider biofilm induced
4228	I64	64 ug/mL FLC	FH1	Missense	1	1519592	G	T	1	0	0.83	0.17	Glu443Asp	orf19.6193	C1_07110W_A	TAF145	Protein similar to S. cerevisiae Taf145p, a component of RNA polymerase II transcription factor TFIID; flucytosine repressed; likely to be essential for growth, based on an insertional mutagenesis strategy
4230	K64	64 ug/mL FLC	FH1	Synonymous	1	150727	A	G	1	0	0.94	0.06		orf19.6027	C1_00790W_A		Ortholog of C. dubliniensis CD36 : Cd36_00740, C. parapsilosis CDC317 : CPAR2_110170, Candida tenuis NRRL Y-1498 : CANTEDRAFT_133175 and Debaryomyces hansenii CBS767 : DEHA2D09724g
4231	L64	64 ug/mL FLC	FH1	Missense	2	776328	A	T	1	0	0.94	0.06	Glu270Asp	orf19.852	C2_03680W_A	SAP98	Glycosyl-phosphatidylinositol-anchored aspartic endopeptidase; regulated by Gcn2p and Gcn4p; expressed only in opaque MTLa/MTLa cells

Strain Number	Lineage Name	Treatment	Progenitor	Chromosome	First Informative Allele	Last Informative Allele	LOH Size	Notes
4052	A1	1 ug/mL FLC	SC5314	5	553838	562410	8572	segmental aneupoidy; i(5L) present
4052	A1	1 ug/mL FLC	SC5314	5	595261	1184123	588862	segmental aneuploidy; i(5L) present
4053	B1	1 ug/mL FLC	SC5314	5	474553	1190928	716375	segmental aneuploidy; i(5L) present; last informative allele is to right telomere. First informative allele is immediately after right copy of the inverted repeat adjacent to CEN5. Same start allele as SC5314 D1.
4055	D1	1 ug/mL FLC	SC5314	5	474553	557773	83220	segmental aneuploidy; i(5L) present; first informative allele is immediately after right copy of the inverted repeat adjacent to CEN5. Same start allele as SC5314 B1.
4055	D1	1 ug/mL FLC	SC5314	6	288292	404711	116419	segmental aneuploidy
4061	J1	1 ug/mL FLC	SC5314	2	0	2232035	2232035	Whole-Chr2 LOH
4061	J1	1 ug/mL FLC	SC5314	R	0	2286239	2286239	Whole-ChrR LOH
4103	P1	1 ug/mL FLC	P75063	6	0	1033530	1033530	Whole-Chr6 LOH
4103	P1	1 ug/mL FLC	P75063	R	0	2286239	2286239	Whole-ChrR LOH
4128	Q64	64 ug/mL FLC	P75063	4	0	1603443	1603443	Whole-Chr4 LOH

In addition to DNA copy number and LOH events, we determined the spectrum of SNVs in coding sequences from each lineage. We identified 57 and 69 high confidence de novo SNVs that reached allele frequencies of 5–100% in the SC5314- and P75063-derived lineages (Table 3). The greatest number of SNVs were in the SC5314 no drug-evolved lineages (33/57, fig. 3*E*) and the P75063 1 μg/ml FLC-evolved lineages (28/69, fig. 3*F*). A majority of all SNVs resulted in nonsynonymous substitutions (30/57 SNVs from SC5314; 45/69 SNVs from P75063). Of the FLC-evolved lineages, more SNVs were acquired at 1 μg/ml than in the other drug concentrations, regardless of genetic background. To ask if any gene functions were enriched within the genes harboring SNVs from all FLC-evolved lineages (1, 8, and 64 μg/ml FLC) we performed gene ontology (GO) analysis. The cellular process “long-chain fatty acid metabolic process” (encompassing genes *ACC1*, *CTF1*, and *POX1-3*) was the only GO term significantly enriched for the FLC-evolved lineages (*P* < 0.05, hypergeometric distribution with Bonferroni Correction).

Next, we filtered for de novo alleles that reached high frequency in each lineage that might explain the evolved phenotypes. For example, the diploid Lineage E_1_ evolved a 4-fold increase in MIC and acquired only one missense allele *ECM25*^Ser467Asn^ at a frequency of ∼54%. *ECM25* encodes a protein involved in cell wall biosynthesis, cell separation and morphogenesis in *C. albicans* and the *S. cerevisiae* ortholog is required for stress-induced cell elongation (Zhang et al. 2008). Lineage E_1_ also acquired multiple aneuploidies of i(5L), Chr3, and Chr7, however, so future work is needed to determine which mutations (alone or in combination) are beneficial. Only three nonsense alleles were identified, in YAK1^Tyr385*^ (P75063-R_8_, 22% frequency), DUT1^Gln56*^ (SC5314-D_64_, 18%), and DOT1^Glu127*^ (P75063-Q_1_, 10%). *YAK1* encodes a serine–threonine protein kinase and inhibition of Yak1 was recently shown to prevent filamentation in *C. albicans* (MacAlpine et al. 2021), which may provide these cells with adaptive benefit in the in vitro evolution experiment where filamentation is not required. Ultimately, linking putative causal mutations to the observed phenotypes will require additional experiments that take the effect of aneuploidy and polyploidy into account as well.

Intriguingly, no SNVs were detected in genes known to cause drug resistance in *C. albicans*. This is in stark contrast to the narrow and recurrent SNVs identified in haploid fungal species like *Saccharomyces cerevisiae, Candida glabrata*, and *Candida auris* during adaptation to similar FLC concentrations (Anderson et al. 2003, 2004; Rybak et al. 2020; Ksiezopolska et al. 2021; Burrack et al. 2022). Although no identical SNVs arose independently in different lineages, several genes acquired SNVs in different lineages (*RIM101*, *DAL5*, *RGD3*, *PXP2*, *TERT*, *orf19.3604*, *orf19.6457*, and *orf19.6970*). However, many of these SNVs encoded synonymous mutations, including a missense and synonymous SNV in *RIM101* from lineage P75063-T_64_ and SC5314-A_1_. The overall pattern is that polyploidy, aneuploidy, and segmental aneuploidy is likely a faster route to adapting to FLC in diploid *C. albicans* isolates and these karyotypic mutations are more likely to repeatedly arise and be selected independently in different lineages than rare adaptive point mutations, at least in the early stages of FLC exposure (Yang, Todd, et al. 2021; Gerstein and Sethi 2022).

In summary, drug concentration dramatically impacts both the phenotypic and genotypic basis of adaptation of lineages from two diverse genetic backgrounds of *C. albicans* evolved for 100 generations. We rationalized that this was due to the relative stress imposed on the cells rather than an inherent property of the specific drug concentration itself, as the two progenitors have the same initial MIC (0.5 μg/ml). We therefore hypothesized that a different progenitor with a higher initial FLC MIC would also acquire a unique mutational signature at a drug concentration near its initial MIC compared with other drug concentrations.

### Mutational Spectrum and Adaptive Potential are Impacted by Initial Fitness in Fluconazole

To test whether a higher initial FLC MIC would alter the spectrum of adaptive mutations, we evolved lineages from clinical isolate FH1, which has an initial FLC MIC of 8 μg/ml (Marr et al. 1997, 1998, 2001). Importantly, isolates with an MIC ≥ 8 μg/ml are defined as clinically resistant due to statistically increased treatment failures (Pfaller et al. 2010, 2011), however why treatments fail is poorly understood. We performed identical in vitro evolution experiments as described above.

**Fig. 4.**
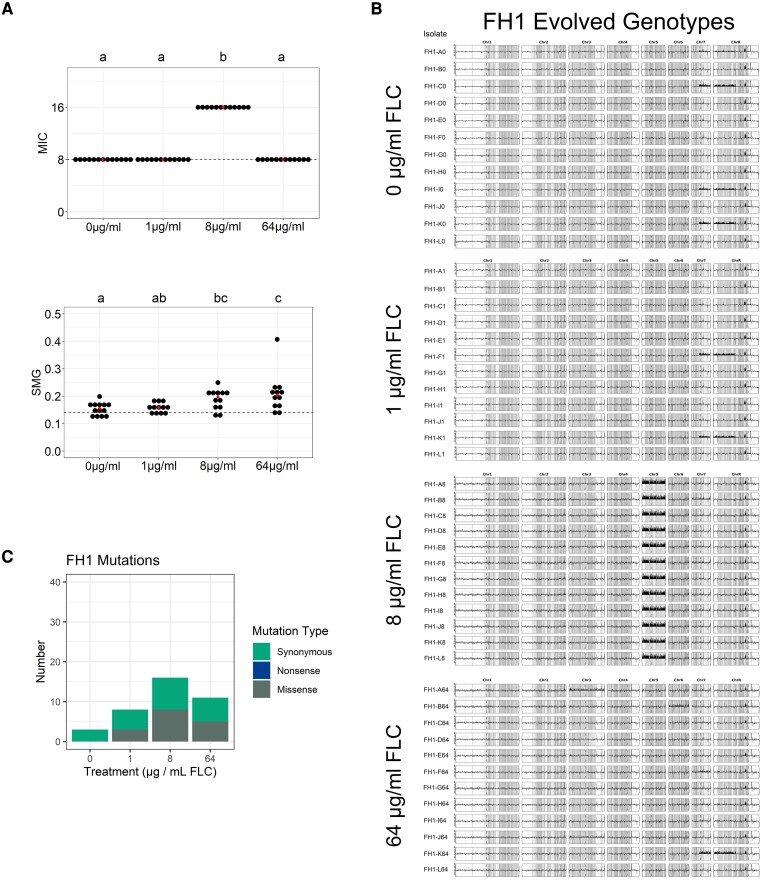
Initial MIC alters the mutational spectrum of evolved lineages. (*A*) Quantification of MIC and SMG values presented as in figure 1. Median MIC and SMG values for each treatment group (diamonds) and of the FLC-sensitive progenitor FH1 (dashed line) for the 48 replicate lineages are plotted in separate panels. Groups not sharing any letter are significantly different (*P* < 0.05, Kruskal-Wallis with Dunn's multiple comparison). The 8 μg/ml treatment group had a significantly higher median MIC than the three other treatment groups. Lineages exposed to 8 μg/ml and 64 μg/ml FLC had a significantly higher SMG than lineages exposed to 0 μg/ml FLC. (*B*) Whole-genome sequence data plotted as in figure 3 with chromosome copy number (y-axis, 1–4 copies) as a function of chromosome position (x-axis, Chr1-ChrR). The ploidy of all FH1-evolved lineages remained 2*N* by flow cytometry (Table 2). FH1 lineages are grouped by drug treatment (0 μg/ml FLC, 1 μg/ml FLC, 8 μg/ml FLC, and 64 μg/ml FLC). All lineages evolved at 8 μg/ml FLC acquired an extra copy (trisomy) of the same Chr5 homolog (Chr5*B*). (C) Frequency and type of single-nucleotide changes identified in the 48 replicate lineages.

Resistance (MIC) and tolerance (SMG) assays were conducted as previously described. No changes in MIC or SMG were observed at 0 μg/ml and 1 μg/ml FLC. Strikingly, the lineages evolved in 8 μg/ml FLC acquired a significantly higher MIC than the three other treatment groups (fig. 4*A*, supplementary fig. S5, Supplementary Material online, *P* < 0.05, Kruskal-Wallis with Dunn's multiple comparison test). Median SMG was minimally but significantly increased in both the 8 μg/ml and 64 μg/ml FLC lineages relative to the 0 μg/ml FLC and 1 μg/ml FLC lineages. This is in sharp contrast to lineages evolved from the other two progenitors that acquired strong tolerance phenotypes at drug concentrations well above their initial MIC and may indicate that adaptation via tolerance is less accessible to this progenitor.

Ploidy analysis and whole-genome sequencing were performed on all 12 FH1 lineages from all four treatment conditions (48 total). All FH1-evolved lineages remained diploid (Table 2), consistent with recent ploidy analysis of FH1 after evolution to 1 μg/ml FLC (Gerstein and Berman 2020). Strikingly, trisomy of Chr5 was observed in all 12 lineages evolved in the presence of 8 μg/ml FLC (fig. 4*B*). Additionally, all 12 lineages acquired an extra copy of the same Chr5 homolog, Chr5B. In the FH1 progenitor, the Chr5B homolog contains a beneficial allele of *TAC1* (*TAC1-7*), and amplification of this homolog was previously observed in an FH1 isolate obtained from an agar plate containing 10 μg/ml FLC (Coste et al. 2006). Two segmental aneuploidies on Chr7 and ChrR were detected at low frequency in multiple evolved lineages from the 0, 1, and 64 μg/ml FLC groups (fig. 4*B*). These segmental aneuploidies were likely present in some of the initial single colony lineages and did not correlate with changes in MIC or SMG. Finally, no LOH was detected in any of the FH1-evolved lineages.

In addition to chromosome amplification, we also identified 38 high confidence de novo SNVs that reached allele frequencies of 5–100% within coding sequences across all FH1 lineages (Table 3). The greatest number of SNVs per treatment was in the 8 μg/ml FLC lineages (fig. 4*C*). Therefore, lineages derived from all three progenitors (FH1, SC5314, and P75063) acquired the most SNVs during adaptation to the drug concentration that was closest to their initial MIC. Only two genes (*ALS2* and *TLO9*) were mutated in different lineages from FH1 or in combination with the SC5314 and P75063 lineages, and both genes represent large gene families. Together, these findings indicate that strain background can significantly influence adaptation, with the initial MIC relative to the environment impacting both mutational spectrum and evolutionary trajectory during FLC treatment.

## Discussion

Higher daily doses of FLC have resulted in longer survival of patients with candidiasis (Schiave et al. 2018). Why this is, and how isogenic *C. albicans* lineages adapt to different physiological concentrations of FLC within host niches remains largely unknown. In this study, we conducted 144 parallel in vitro evolution experiments to examine the impact of drug concentration on the genotypic and phenotypic basis of adaptation. We found that drug concentration dramatically impacts both drug response phenotypes (resistance or tolerance) and the spectrum of mutations acquired across three different genetic backgrounds. In general, concentrations of FLC that were at or two times above the MIC (near-MIC) of the progenitor selected for lineages that have significantly increased drug resistance, whereas higher concentrations of FLC (supra-MIC) selected for lineages with significantly increased drug tolerance. The acquisition of recurrent and distinct whole-chromosome and segmental aneuploidy was extremely prevalent in drug concentrations near the initial MIC, with the specific evolved karyotype linked to both progenitor strain and drug environment. Adaptation to FLC did not occur in either the absence of drug or in drug concentrations below that of the progenitor's starting MIC. These findings highlight that the initial MIC of a clinical isolate can dramatically alter how the isolate adapts to FLC and that different concentrations of the same drug select for different evolutionary trajectories. Specifically, we observed that selection seems to act either to increase FLC resistance or FLC tolerance, suggesting phenotypic improvement in these traits represents distinct peaks on the fitness landscape. These findings have broad implications for antifungal drug therapies and clinical best practices and could underlie why some treatment regimens fail. Current and future screens for novel antifungal drugs should take multiple drug concentrations into effect to assess efficacy and to understand how differing concentrations may impact fitness outcomes and potential treatment failure.

We found that lineages from three progenitors evolved in near-MIC concentrations of FLC increased in resistance to FLC. Lineages from all three progenitors exposed to supra-MIC concentrations significantly increased their tolerance to FLC, though the degree of tolerance was much lower in the third progenitor (FH1, which has an ancestrally higher MIC). We did not identify a correlation between genome size, appearance of aneuploidies, or SNVs in specific genes and the tolerance of a lineage, indicating that many different genotypes or epistatic interactions may drive the drug response phenotypes. The rapid increase in tolerance (but not resistance) at supra-MIC levels, as well as a lack of correlation with evolved genome size is consistent with a recent evolution study that evolved lineages of *C. albicans* to supra-MIC levels of the azole Posaconazole (Kukurudz et al. 2022). Although the drivers of antifungal drug tolerance remain largely unknown, genes involved in core stress responses likely play a role (Cowen et al. 2014; Rosenberg et al. 2018; Berman and Krysan 2020). Cells exposed to supra-MIC concentrations of antifungal drug may be able to slow growth and division enough to preserve viability, whereas lower concentrations of antifungal drugs may still allow cells to progress through the cell cycle leading to the acquisition of segmental aneuploidy or point mutations that result in *bona fide* resistance. A small number of evolved lineages acquired both high tolerance and high resistance (e.g., SC5314-G_1_ and P75063-N_8_) and will be valuable for future work that aims to tease apart the molecular mechanism driving both phenotypes.

Karyotypic variation (i.e., whole chromosome, isochromosome, and segmental aneuploidy) were observed in all three different progenitor backgrounds following FLC evolution. By comparing multiple drug concentrations, we found that a different suite of genomic changes occurred when *C. albicans* was evolved to FLC concentrations near the initial MIC of the progenitor strains, compared with concentrations above the MIC. We propose that the observed differences in evolved genotype are due to the relative stress on the cells rather than an inherent property of the specific drug concentration. Our results suggest that the critical distinction is not drug concentration per se, but a physiological breakpoint where cells experience one degree of stress near their initial MIC, and a different degree of stress below and above their initial MIC.

Whole-chromosome aneuploidy of Chr3, Chr5, Chr6, and ChrR was the most common across all evolved lineages. Aneuploidy (whole and partial chromosome) has been observed in most fungal pathogens, including in isolates obtained directly from patients and/or during experimental evolution in the presence of antifungal drugs (Marichal et al. 1997; Sionov et al. 2010; Ngamskulrungroj et al. 2012; Demers et al. 2018; Ksiezopolska et al. 2021; Yang, Lu, et al. 2021; Burrack et al. 2022). Some aneuploidies were more common from some progenitor strains and in response to some environments. The most striking example of this phenomenon was the increase in MIC and recurrent amplification of Chr5B in all 12 lineages derived from FH1 during adaptation to 8 μg/ml FLC (fig. 4), which was not observed during adaptation to lower *or* higher concentrations of drug in FH1. This suggests that there may be a fitness tradeoff to the beneficial effects of Chr5B aneuploidy in the FH1 background at both lower and higher concentrations of FLC. FH1 is the first in a series of isolates obtained from a bone marrow transplant patient before initiation of antifungal therapy including FLC (Marr et al. 1997, 1998). Later isolates from this patient (FH2-FH9) are related to FH1 and several independently acquired amplification of Chr5B on an isochromosome or homozygosis of the *TAC1-7* allele on Chr5B (Coste et al. 2006; Selmecki et al. 2006, 2008; Abbey et al. 2014). Furthermore, both Chr5A and Chr5B aneuploidy was observed in SC5314 and P75063 lineages evolved in different drug concentrations. Finding extremely parallel aneuploidy is exciting from a clinical viewpoint. An “evolutionary trap” approach to extend the life of existing antifungal drugs was proposed by Rong Li and colleagues where treatment with a single antifungal drug selects for a genotype that can be targeted by a secondary drug. Their screen identified the FDA-approved drug pyrvinium that caused increased killing of *C. albicans* cells that had an i(5L) aneuploidy compared with wildtype cells (Chen et al. 2015). Our data support that this evolutionary trap may be incredibly effective at near-MIC FLC concentrations, where we observed recurrent alterations of Chr5 in all three genetic backgrounds.

In addition to the isochromosome of Chr5, segmental aneuploidy on Chr1, Chr3, and Chr4 was identified in six lineages from P75063. These segmental aneuploidies consistently amplify large regions of a chromosome and can form via an unstable dicentric chromosome that progresses through successive rounds of breakage-fusion-bridge cycles that are repaired via non-allelic homologous recombination between long inverted repeat sequences (Todd and Selmecki 2020). Several of these segmental aneuploidies were shown to be sufficient to confer FLC resistance (Selmecki et al. 2008, 2009; Todd et al. 2019). The experiments here are the first to clarify that segmental aneuploidies recurrently and predominantly form only at FLC concentrations that are near the MIC of the original progenitor isolate, and furthermore suggest that the propensity to acquire segmental aneuploidy is background-dependent. We also found that drug concentration impacts the frequency of i(5L) formation. i(5L) was only observed in lineages evolved in 1 μg/ml FLC from progenitor SC5314 (the *C. albicans* reference strain; 5/6 lineages) and P75063 (2/6 lineages). Interestingly, the centromere-specific histone H3 variant Cse4/CENP-A is depleted from the centromere during growth in 10 μg/ml FLC, which increases the rate of chromosome missegregation in the SC5314 background (Brimacombe et al. 2019). Therefore, it is tempting to speculate that at low concentrations of FLC (1 μg/ml), Cse4 binding is still sufficient to promote dicentric chromosome formation and breakage-fusion-bridge cycles that can promote segmental aneuploidies, but at higher FLC concentrations, including 8 μg/ml, 10 μg/ml, and 64 μg/ml FLC, centromeres are more destabilized and whole-chromosome aneuploidy and polyploidization are observed more frequently.

In addition to finding the evolution of distinct aneuploidies, at all drug concentrations we also found multiple lineages from SC5314 and P75063 where polyploid cells swept the population within 100 generations of evolution. In *C. albicans*, acute treatment (8 h) with 10 μg/ml FLC can induce polyploidization in ∼20% of cells in a population (Harrison et al. 2014). These polyploid “timera” cells form after failed cytokinesis and can give rise to highly aneuploid daughter cells with increased MICs (Harrison et al. 2014). Likewise, *Cryptococcus neoformans* polyploid “titan” cells exposed to FLC rapidly produced highly aneuploid daughter cells with increased fitness in the presence of FLC (Gerstein et al. 2015). In a large-scale parallel in vitro evolution experiment, 20 diverse *C. albicans* clinical isolates were evolved for 100 generations in a single concentration of FLC (1 μg/ml) (Gerstein and Berman 2020). In parallel to results here, changes in genome size were pervasive in all backgrounds except for those whose initial MIC was higher than 1 μg/ml (Gerstein and Berman 2020). Here we found that FH1 remains diploid at all drug concentrations tested, whereas polyploidization occurred more frequently in P75063 lineages than in SC5314 lineages (fig. 2). This is in contrast to whole-chromosome aneuploidy, where SC5314 lineages had nearly four times the number of aneuploidy events than P75063. Aneuploid chromosomes in SC5314 may be a result of transient polyploidization followed by concerted chromosome loss events resulting in highly aneuploid cells (Bennett and Johnson 2003; Hickman et al. 2015; Gerstein et al. 2017; Avramovska and Hickman 2019). Polyploidization can have significant impact on the rate and dynamics of subsequent adaptive events, including acquisition of beneficial point mutations and aneuploid chromosomes (Scott et al. 2017). *S. cerevisiae* polyploid cells acquire significantly more point mutations, segmental, and whole-chromosome aneuploidies than diploid cells during adaptation to low carbon environment (Selmecki et al. 2015; Scott et al. 2017). Additionally, the fitness effect of a given mutation, including aneuploidy, can change with polyploidy and can reveal beneficial effects that do not provide a similar benefit to isogenic diploids (Selmecki et al. 2015). Using whole-genome sequencing we also found polyploidy was frequently associated with chromosome aneuploidy, and interestingly, all the polyploid isolates that we randomly selected for sequencing contained amplification of ChrR (fig. 3*A* and *B*). Further analysis is needed of the trajectory of polyploid cells over the course of evolution.

Surprisingly, no SNVs were observed in well-known drug resistance factors in the timeframe of our experiments with initially diploid *C. albicans*. During adaptation to FLC in vitro, haploid yeast species including *S. cerevisiae*, *C. glabrata*, and *C. auris* acquired mutations that are recurrent and narrow in spectrum, including mutations in *PDR1*, *ERG3*, *ERG11*, *UPC2*, *TAC1, MDR1*, *CDR1*, *CDR2* (Anderson et al. 2003, 2004; Rybak et al. 2020; Ksiezopolska et al. 2021; Burrack et al. 2022). The rate and spectrum of acquired point mutations is impacted by cellular ploidy in many environments through dominance as well as differences in effect size (Gerstein et al. 2006; Gerstein 2013; Selmecki et al. 2015; Buskirk et al. 2017; Scott et al. 2017; Fisher et al. 2018; Marad et al. 2018). Elegant bulk-segregant fitness analysis in *S. cerevisiae* found that diploid populations contain fewer driver mutations and more hitchhiker mutations relative to haploid populations evolved in the same environment, and that all beneficial mutations in diploids were dominant or overdominant (Aggeli et al. 2021). Determining the impact of *C. albicans* ploidy on the rate and mechanism of adaptation to different drug concentrations may be possible in the future, including a direct comparison of isogenic haploid, diploid and tetraploid *C. albicans*, however stable haploid and tetraploid lineages of *C. albicans* currently do not exist (Hickman et al. 2015; Selmecki et al. 2015).

### Future Directions

Available antifungal drugs are limiting (Revie et al. 2018; Perfect et al. 2022), and it is critical to understand the mechanisms by which resistance and tolerance evolve. Considerable effort has been extended to characterize genic mutations. The same effort has not yet been extended to understanding the mechanisms that underlie aneuploidy-associated antifungal resistance, despite the observed high frequency of aneuploidy in clinical and experimental fungal isolates. We previously showed that the gene copy numbers of *ERG11* and *TAC1* on the left arm of Chr5 are sufficient for FLC resistance in an i(5L) strain, and there is a linear correlation between their combined gene copy-numbers and FLC MIC (Selmecki et al. 2008). For other aneuploidies the mechanism is less clear. In general, aneuploidy alters cell physiology in ways that may promote antifungal tolerance or resistance. Aneuploid budding yeast cells exhibit increased plasma-membrane stress and impaired endocytosis that may alter metabolic and proteomic homeostasis leading to altered fitness states during periods of cellular stress (Torres et al. 2007; Pavelka et al. 2010; Tsai et al. 2019). Future studies are needed to comprehensively determine which gene(s) on an amplified chromosome are under selection across diverse genetic backgrounds/fungal species. Development of CRISPR tools that amplify gene expression of every gene on an aneuploid chromosome or region will help determine what genes in an amplified region are under selection during antifungal treatment (Uthayakumar et al. 2020). These molecular tools may further reveal the mechanisms that are under selection at specific drug concentrations, and in different genetic backgrounds, including allele-specific phenotypes that are under selection in these distinct genetic backgrounds.

## Materials and Methods

### Yeast Strains and Culture Conditions

All strains used in this study are described in Table 1. Strains were stored at −80°C in 20% glycerol. Strains were cultured in YPAD medium (yeast extract, peptone, and 2% dextrose) supplemented with 40 μg/ml adenine and 80 μg/ml uridine. To start the in vitro evolution experiment, the FLC-susceptible progenitor clinical isolates SC5314 and P75063 were plated for single colonies on YPAD + 2% agar plates directly from the −80°C. Plates were left to incubate for 48 h in a 30°C incubator.

### *In Vitro* Evolution Experiment

The FLC-susceptible progenitor clinical isolates (SC5314, P75063, and FH1) were plated for single colonies onto YPAD + 2% glucose agar medium and incubated for 48 h at 30°C. Twelve single colonies from each progenitor isolate were selected at random and suspended in 1 ml of sterile liquid YPAD medium and incubated overnight at 30°C. After growth overnight, each single colony liquid suspension was diluted 1:1000 and used to start four independent lineages, defined as treatment groups (YPAD only, YPAD + 1 μg/ml FLC, YPAD + 8 μg/ml FLC, and YPAD + 64 μg/ml FLC) in deep-well 96-well plates. Plates were sealed with Breathe EASIER tape (Electron Microscopy Sciences) and placed in a humidified chamber for 72 h at 30°C. Every 72 h, cells were carefully resuspended and transferred to fresh medium containing the same concentration of FLC to a final cell dilution of 1:1000. In total, 10 transfers were conducted. After the final transfer, cells were collected for storage at −80°C, genomic DNA isolation, and for phenotypic analyses.

### Microdilution MIC and SMG Assays

The microwell broth dilution assay was used to determine both the MIC_50_ and SMG for each lineage put through the in vitro evolution experiment. Lineages evolved in FLC during the in vitro evolution experiment were inoculated from a −80°C freezer into fresh liquid YPAD medium supplemented with 1 μg/ml FLC and grown for 16 h in a 30°C shaking incubator. Lineages evolved in the absence of FLC during the in vitro evolution experiment were inoculated into fresh liquid YPAD (with no added FLC) and grown for 16 h in a 30°C shaking incubator. From these cultures, cells were inoculated into a 96-well plate containing 180 μl of a 0.5X dextrose YPAD medium with a 2-fold serial dilution of FLC or a no-drug control to a final cell dilution of 1:1000 and a final volume of 200 μl. Cells were incubated at 30°C in a humidified chamber and OD_600_ readings were taken at both 24 and 48 h post inoculation; cells were resuspended by pipette prior to reading. The MIC_50_ of each lineage was determined to be the concentration of FLC at which ≥50% of growth was inhibited when compared with the no-drug control. Supra-MIC growth (SMG) was calculated by taking the average OD_600_ value of the wells above the 24 h MIC_50_ at 48 h and dividing by the OD_600_ in the no-drug control well (Rosenberg et al. 2018).

### Growth Curve Analysis

For growth curve analysis, cells were cultured overnight in 3 mL YPAD liquid culture, diluted to an OD_600_ value of 0.1 and aliquoted into a 96-well plate with either YPAD or YPAD + 1 μg/ml FLC. Cultures were grown at 30°C with constant dual-orbital agitation in a Biotek Epoch plate reader, and OD measurements were taken every 15 min for 30 h. All growth curves were performed in biological triplicate. Growth curves (mean OD by time) were plotted with R and the tidyverse package (Wickham et al. 2019). Summary statistics for growth curves, including the fitted logistic models and areas under the fitted curve, were calculated using default parameters with the R package Growthcurver (Sprouffske and Wagner 2016). Significant differences were determined using the area under the curve for each lineage and treatment (three replicates in each group, *P* < 0.05, ANOVA with Tukey post-hoc test).

### Illumina Whole-Genome Sequencing

Genomic DNA was isolated using a phenol-chloroform extraction as previously described (Selmecki et al. 2006). Libraries were prepared using either the Illumina Nextera XT DNA Library Preparation Kit or the Nextera DNA Flex Library Preparation Kit. Adaptor sequences and low-quality reads were removed using Trimmomatic (v0.33 LEADING:3 Trailing:3 SLIDINGWINDOW:4:15 MINLEN:36 TOPHRED33) (Bolger et al. 2014). Trimmed reads were mapped to the *C. albicans* reference genome (A21-s02-m09-r08) from the *Candida* Genome Database (CGD) (http://www.candidagenome.org/download/sequence/C_albicans_SC5314/Assembly21/). Reads were mapped using BWA-MEM (v0.7.12) with default parameters (Li 2013). PCR duplicates were removed using Samtools (v0.1.19) (Li et al. 2009), and realigned around predicted indels using the Genome Analysis Toolkit (RealignerTargetCreator and IndelRealigner, v3.4–46) (McKenna et al. 2010). The indel realignment step was skipped for FH1 variant detection, as this step is not required with mutect2. All new WGS Illumina data have been deposited in the National Center for Biotechnology Information Sequence Read Archive (SRA) database under PRJNA741683; previously published data are available under PRJNA510147 and PRJNA613282 (Table 1).

### Visualization of Aneuploid Chromosomes

Aneuploidies were visualized using the Yeast Mapping Analysis Pipeline (YMAP, v1.0) (Abbey et al. 2014). BAM files aligned to the SC5314 reference genome (A21-s02-m09-r08) were uploaded to YMAP and read depth was determined and plotted as a function of chromosome position. Read depth was corrected for both chromosome-end bias and GC-content.

### Identification of LOH Events

Preliminary identification of LOH events was conducted using aligned Illumina reads and YMAP plots generated above. YMAP plots for each lineage (e.g., SC5314 A_0_, A_1_, A_8_, A_64_) were visually compared with each other to look for regions underwent homozygosis, based on heterozygosity in the other three lineages at the same region. Approximate LOH boundaries were identified from YMAP GBrowse allele ratio tracks, and confirmed by visual inspection in IGV (IGV, v2.8.2) (Thorvaldsdottir et al. 2013). An LOH event was defined as a transition from at least four consecutive heterozygous alleles to four consecutive homozygous alleles and vice versa. Heterozygous alleles had an alternate allele frequency of at least 20%, with at least a read depth of 10, and forward and reverse strands supporting the alternate allele. The position of the first and last informative homozygous alleles (LOH breakpoints) was recorded along with the lineage in which this occurred and the length of the LOH event (last informative allele position minus the first informative allele position, Table 3). If the LOH breakpoints were within 5000 bp of the start or end of the chromosome sequence, the breakpoint was considered to be to the telomere end, and the first or last nucleotide position of the chromosome was recorded. If both breakpoints were to the ends of a chromosome, the LOH event was denoted as a whole-chromosome LOH.

### Variant Detection

Variant detection was conducted using the aligned, sorted, PCR duplicate-removed BAM files (see Illumina whole-genome sequencing above). Variants were detected using the Genome Analysis Toolkit (mutect, v2.2-25-g2a68eab for SC5314 and P75063; mutect2, v4.1.2.0 for FH1). Variants were annotated using SnpEff (v4.3) using the SC5314 reference genome fasta file (A21-s02-m09-r08) and gene feature file (http://candidagenome.org/download/chromosomal_feature_files/C_albicans_SC5314/archive/). Variants were filtered using SnpSift to select for coding mutations of missense, nonsense, synonymous, start_loss, or stop_loss type (Cingolani et al. 2012). Parental variants were removed using VCF-VCF Intersect (v1.0) on the Galaxy Web Platform for SC5314 and P75063 variant calls; this step was integrated into mutect2 and so was not manually done for FH1 variant calls (Afgan et al. 2016).

Identification of de novo variants (variants that arose during the evolution experiment, and therefore were likely not present in a significant proportion of the initial progenitor population) required additional filtering steps. First, variants were kept if they satisfied the following criteria: at least five reads contained the alternate allele; at least one read in the forward and reverse direction contained the variant; the variant was not located in a repetitive region, such as the MRS or ribosomal subunits (Todd and Selmecki 2020). Then, variants present in the initial progenitor were defined as those that fit either criteria: A) an identical variant found in at least half of the sequenced lineages of the no drug control experiments (i.e., 3/6 lineages for SC5314 and P75063, or 6/12 lineages for FH1); or B) variants that were present in all four drug treatments of a given lineage. These progenitor variants were removed from the de novo mutation list. All variants were verified visually using the Integrative Genomics Viewer (IGV, v2.8.2) (Thorvaldsdottir et al. 2013). Mutations were then annotated with gene descriptions from the CGD (Table 3).

### Gene Ontology Analysis

GO Term Finder analysis (Boyle et al. 2004) was conducted on the set of all genes with SNVs, from drug-treated (1, 8, 64 μg/ml FLC) strains with progenitors SC5314 or P75063 for Process, Function, and Component Ontology, on the CGD (accessed August 31, 2022). The only significant cluster detected is for the GO term long-chain fatty acid metabolic process (*P* < 0.05, hypergeometric distribution with Bonferroni Correction). Similar analyses were conducted with the set of all genes with SNVs from drug-treated (1, 8, 64 μg/ml FLC) lineages with progenitors SC5314, P75063, and FH1, but no significant terms were identified.

## Supplementary Material

msad009_Supplementary_Data

## Data Availability

The data underlying this article are available in the article, in its online supplementary material, and will be shared on reasonable request to the corresponding author.
