# Supplementary material for: Antifungal Drug Concentration Impacts the Spectrum of Adaptive Mutations in Candida albicans
Source: Mol Biol Evol. 2023 Jan 17;40(1):msad009. doi: 10.1093/molbev/msad009 (PMC9887641; doi:10.1093/molbev/msad009)
Supplement: msad009_Supplementary_Data [file msad009_supplementary_data.pdf]

SUPPLEMENTARY FIGURES

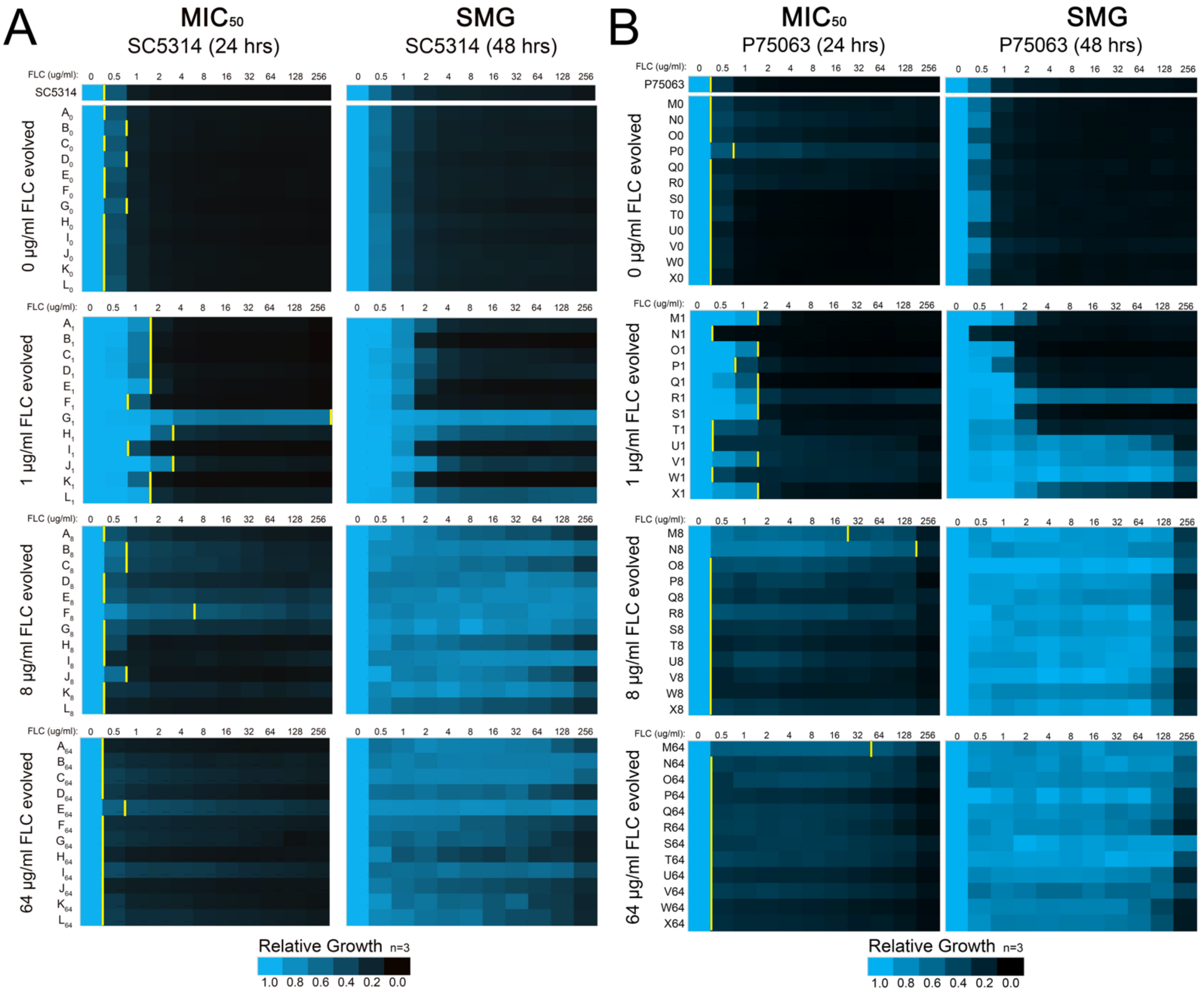

Supplementary fig.S1. Drug response phenotypes MIC and SMG quantified using 96-well plate assay for growth in increasing concentrations of fluconazole.

Heat maps of growth (OD_{600}) at 24 (left) and 48 hours (right) in two-fold increasing concentrations of FLC for **(A)** SC5314 and **(B)** P75063 lineages. The drug concentration at which 50% of the growth was inhibited (MIC_{50}) at 24 hours is denoted with a yellow line. Supra-MIC growth (SMG), a measurement of antifungal drug tolerance, was calculated as the average growth at 48 hours above the MIC at 24 hours, divided by the lineage growth at 48 hours in no drug (see Materials and Methods). Each heat map represents the average of three independent MIC or SMG assays.

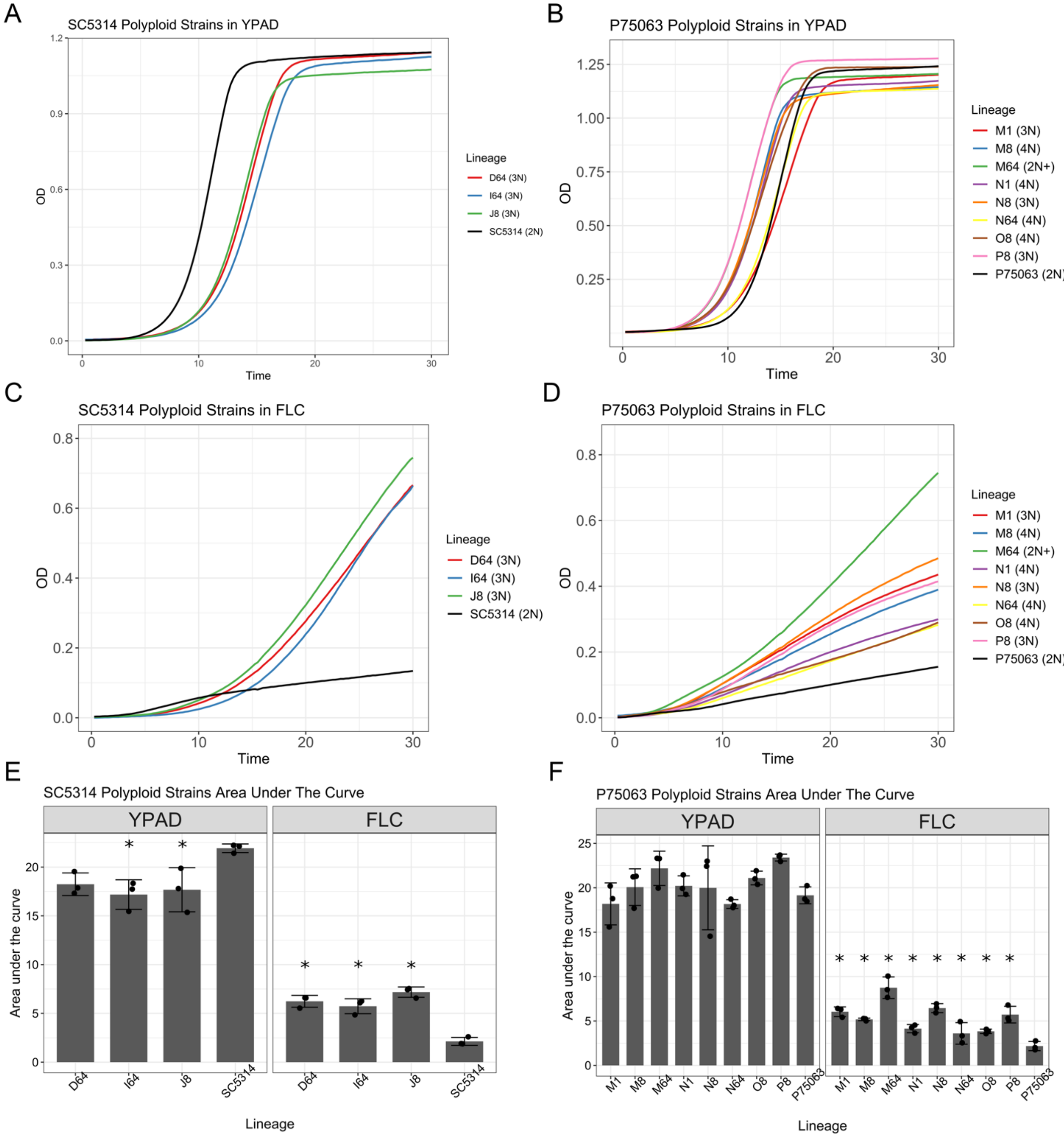

Supplementary fig.S2: Polyploid lineages have significantly improved growth rates in the presence of FLC relative to their diploid progenitors. Plots of mean optical density (OD) in 0µg/ml FLC (YPAD) for polyploid **(A)** SC5314 and **(B)** P75063 lineages and their progenitor. Plots of mean OD in 1µg/ml FLC for polyploid **(C)** SC5314 and **(D)** P75063 lineages and their progenitor. All growth curves were performed in biological triplicate for 30 hours. Plots of area under the curve of **(E)** SC5314 and **(F)** P75063 lineages, in YPAD or FLC treatment. Dots represent the area under the curve for each replicate. Bars represent the mean area under the curve for the group, and error bars represent 1 SD from the mean. Significant differences in mean AUC for each lineage compared to its progenitor (SC5314 or P75063) are marked with an asterisk ($p<0.05$, ANOVA with Tukey post-hoc test).

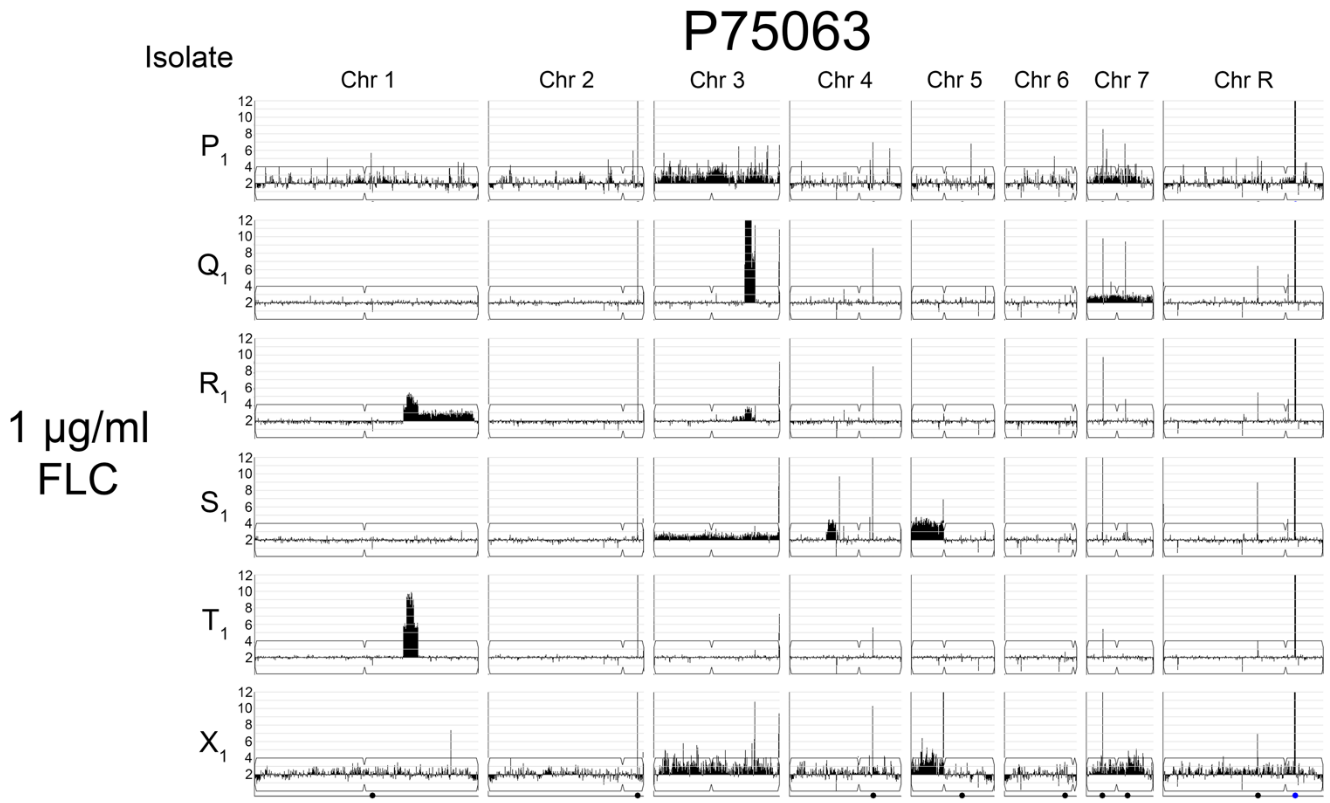

Supplementary fig.S3: Segmental aneuploidies are identified in P75063 derived isolates evolved in 1 µg/ml FLC. Whole genome sequence data of the P75063 1µg/ml FLC-evolved lineages plotted as the log2 ratio and converted to chromosome copy number (y-axis, 1-12 copies) as a function of chromosome position (x-axis, Chr1-ChrR) as in Figure 1. The segmental aneuploidies located on Chr1 (R_1 & T_1), Chr3 (Q_1 & R_1), and Chr4 (S_1) amplify large regions of the genome up to more than 12 copies per genome and are flanked by multiple, long inverted repeat sequences (Todd and Selmecki 2020).

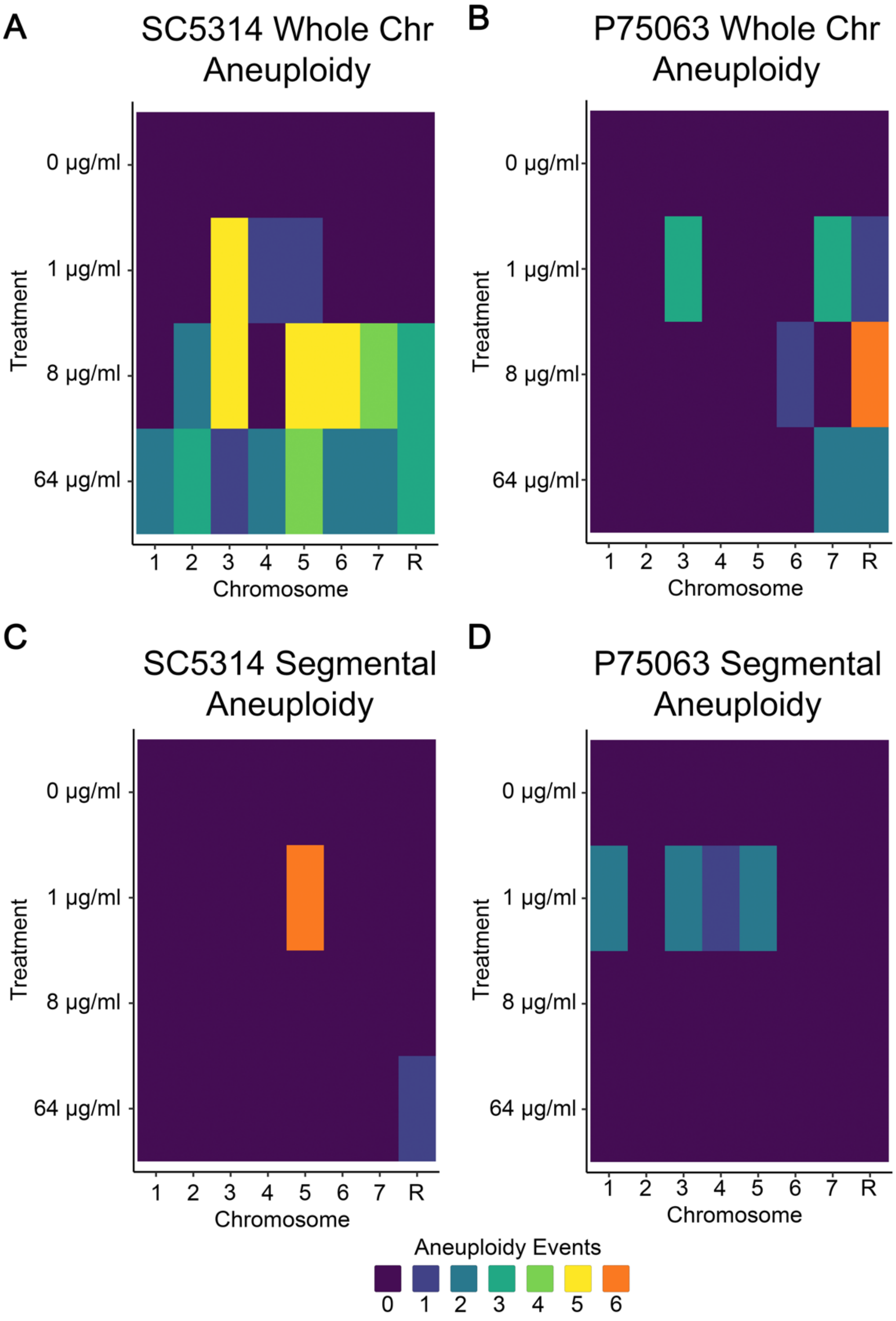

Supplementary fig.S4: Segmental aneuploidies predominantly occur at 1 µg/ml FLC. Heat maps representing the number of whole chromosome aneuploidy events by chromosome for SC5314- **(A)** and P75063-derived **(B)** lineages. Whole chromosome aneuploidies are observed at all drug concentrations tested except the no drug control. Heat maps representing the number of segmental aneuploidy events by chromosome for lineages derived from SC5314 **(C)** and P75063 **(D)**. Segmental aneuploidies were primarily observed in lineages evolved in 1µg/ml FLC.

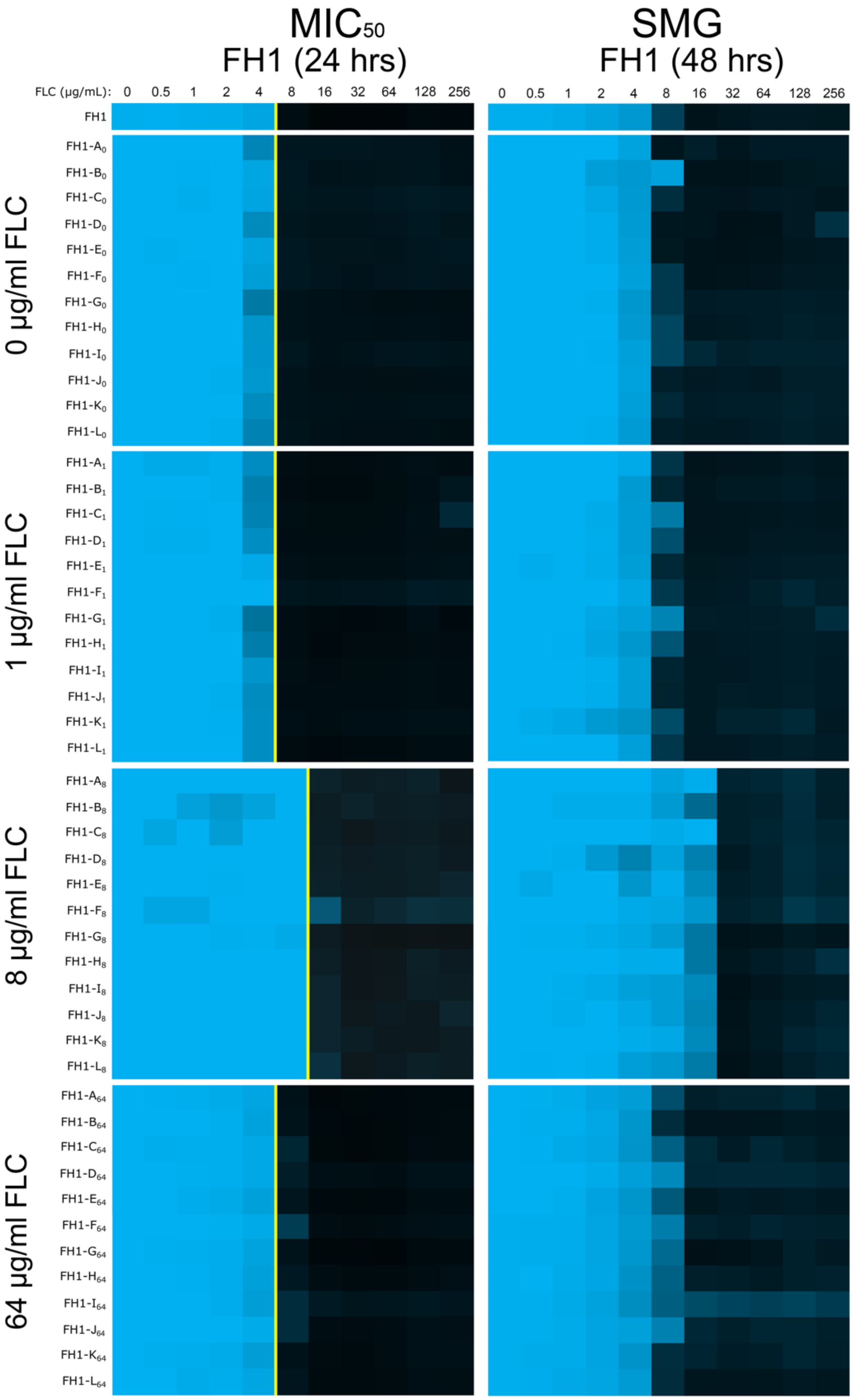

Supplementary fig. S5. Initial MIC alters the mutational spectrum of evolved lineages.

Heat map of FH1-derived lineage growth (OD_{600}) at 24 (left) and 48 hours (right) in two-fold increasing concentrations of FLC. The MIC_{50} at 24 hours is denoted with a yellow line and Supra-MIC growth (SMG) at 48 hours are indicated as in Figure 1. Each heat map represents the average of three independent MIC or SMG assays.
